# Occlusal stabilization splint for patients with temporomandibular disorders: Meta-analysis of short and long term effects

**DOI:** 10.1371/journal.pone.0171296

**Published:** 2017-02-06

**Authors:** Jovana Kuzmanovic Pficer, Slobodan Dodic, Vojkan Lazic, Goran Trajkovic, Natasa Milic, Biljana Milicic

**Affiliations:** 1 Department for Medical Statistics and Informatics, School of Dental Medicine University of Belgrade, Belgrade, Serbia; 2 Department of Prosthodontics, School of Dental Medicine University of Belgrade, Belgrade, Serbia; 3 Department for Medical Statistics and Informatics, School of Medicine University of Belgrade, Belgrade, Serbia; University of Washington, UNITED STATES

## Abstract

**Background:**

Psychological discomfort, physical disability and functional limitations of the orofacial system have a major impact on everyday life of patients with temporomandibular disorders (TMDs). In this study we sought to determine short and long term effects of stabilization splint (SS) in treatment of TMDs, and to identify factors influencing its efficacy.

**Methods:**

MEDLINE, Web of Science and EMBASE were searched for randomized controlled trials (RCTs) comparing SS to: non-occluding splint, occlusal oral appliances, physiotherapy, behavioral therapy, counseling and no treatment. Random effects method was used to summarize outcomes. The effect estimates were expressed as odds ratio (OR) or standardized mean difference (SMD) with 95% confidence interval. Subgroup analyses were carried out according to the use of Research Diagnostic Criteria (RDC/TMD) and TMDs origin. Strength of evidence was assessed by GRADE. Meta-regression was applied.

**Results:**

Thirty three eligible RCTs were included in meta-analysis. In short term, SS presented positive overall effect on pain reduction (OR 2.08; p = 0.01) and pain intensity (SMD -0.33; p = 0.02). Subgroup analyses confirmed SS effect in studies used RDC/TMD and revealed its effect in patients with TMDs of muscular origin. Important decrease of muscle tenderness (OR 1.97; p = 0.03) and improvement of mouth opening (SMD -0.30; p = 0.04) were found. SS in comparison to oral appliances showed no difference (OR 0.74; p = 0.24). Meta-regression identified continuous use of SS during the day as a factor influencing efficacy (p = 0.01). Long term results showed no difference in observed outcomes between groups. Low quality of evidence was found for primary outcomes.

**Conclusion:**

SS presented short term benefit for patients with TMDs. In long term follow up, the effect is equalized with other therapeutic modalities. Further studies based on appropriate use of standardized criteria for patient recruitment and outcomes under assessment are needed to better define SS effect persistence in long term.

## Background

Psychological discomfort, physical disability and functional limitations of the orofacial system have a major impact on everyday life of patients with temporomandibular disorders (TMDs) [1–3]. Epidemiological studies indicate that approximately 10% to 15% of the general population has TMDs; while 5% of the respondents require therapy [4, 5]. The highest prevalence of TMDs is found in subjects between 18 and 45 years of age and is more common in women [6]. The causes of TMDs are not always clearly defined, nor it is known if they originate from the joint structures or muscles, thus influencing decision making in establishing proper diagnosis [7, 8]. Etiological factors are numerous, insufficiently studied, and may alone or in combination contribute to the development of TMDs [9, 10].

A popular dispute regarding occlusal treatments in treating TMDs has an everlasting history and does not seem to be calming down. The fact that nearly 100 million people have TMDs in the USA [11] and that approximately 3 million splints are made per year, at a cost of $990 million only in the United States [12], demonstrate the need for further research of this topic. American Association for Dental Research (AADR) has given a policy statement [13], that managing TMDs should be evidence-based and aiming to provide therapy with the greatest potential for long term symptoms relief. There are many different therapeutic options that can eliminate the symptoms in muscles and jaw joints, and these include the usage of occlusal oral appliances, pharmacological therapy, physical therapy, cognitive-behavioral therapy (CBT), counseling and self-care management or its combinations. One of the preferred therapies for treating patients with TMDs is stabilization splint (SS), a flat occlusal plate made of hard acrylic or polycarbonate material. SS is designed to promote occlusal stability[14] and decrease muscles tension [15].

Conducted randomized controlled trials (RCTs) assessing TMDs presented conflicting results [16–21], while gathered data in systematic reviews didn’t provide clear evidence whether the use of SS is justified in treating TMDs [7, 12, 22–26]. More importantly, approaches used for evaluation of SS in published reviews differ significantly. Several meta-analyses evaluated the efficacy of SS versus individual treatment [12, 24, 25]. Fricton at al. presented results in favor of SS, but pointed out limitations regarding the type of TMDs origin and duration of therapy [24]. Al Ani et al. provided weak evidence of SS efficacy for muscular pain dysfunction syndrome in short and long term [25]. Ebrahim et al. investigated occlusal appliances versus minimal treatment, using different designs of splint, and found important difference between these groups, but they haven’t followed the effect of SS in time [12]. Main reasons that undermined validity of mentioned meta-analyses occurred due to large heterogeneity of included populations and type of TMDs origin, lack of using strict criteria such as established Research Diagnostic Criteria for Temporomandibular Disorders (RDC/TMD or DC/TMD) [27, 28] for studies inclusion [1, 24, 26], and inconsistent assessment of follow up period [12, 24]. Lack of identified factors influencing SS efficacy is also present in the literature [1, 23, 24]. In this study we sought to determine short and long term effects of SS through systematic review and meta-analysis of all eligible RCTs using SS as an intervention, to assess these findings on studies used only RDC/TMD criteria, and to identify factors influencing SS efficacy in reducing signs and symptoms of TMDs.

## Materials and methods

### Search methods for identification of studies

The search for relevant studies in any language was conducted up to October 2016. The following databases were searched individually but revised appropriately for each database: MEDLINE (1966 to the present), Web of Science (1980 to the present) and EMBASE (1966 to the present). Journals considered relevant for TMDs were examined to identify the primary studies. Also, references from primary studies, reviews and systematic reviews were looked into for relevant data. The search strategy used a combination of controlled vocabulary (MESH) and free text terms (controlled vocabulary is given in upper case type and free text terms in lower case): TEMPOROMANDIBULAR JOINT DISORDERS OR temporomandibular disorders OR CRANIOMANDIBULAR DISORDERS OR myofacial pain OR myofascial pain OR orofacial pain OR FACIAL PAIN OR HEADACHE AND stabilization splint OR OCCLUSAL SPLINTS OR occlusal appliance OR splint therapy (S1 File). The process of reporting was conducted using PRISMA Statement (Preferred reporting items for systematic review and meta-analysis protocols) [29] (S1 Checklist).

### Criteria for considering studies for this review

#### Types of studies

All RCTs or quasi-randomized controlled trials in which SS was compared to different control group were selected for this meta-analysis. Abstracts were not considered in the study.

#### Studied population

TMD patients with more than one of following symptoms or signs were included as studied population: myofascial pain and /or pain in the TMJ, myofascial pain and/or pain in the TMJ on palpation, muscles tenderness, limitation or deviation in mandibular range of motion, limited mouth opening with/ without reduction, presence of sound effects in TMJ, headache or earache. Patients with systemic diseases and comorbidities were excluded. Patients who had unsuccessfully undergone splint therapy or other TMD treatments in the past were not excluded.

#### Types of interventions

SS (Michigan splint, Tanner appliance, the Fox appliance, centric relation appliance) compared to control group including any of following treatments was evaluated: non-occluding splint, occlusal oral appliances, physical therapy, behavioral therapy, minimal treatment group (exercise and counseling) and no treatment. Combinations of treatments were also analyzed.

#### Types of outcome measures

Outcome variables were evaluated in short (≤ 3 months) and long term follow up (>3 months). The primary outcomes that were analyzed were:

1. Pain reduction measured categorically and
2. Pain intensity estimated by any recognized validated pain scale: visual analogue scale (VAS), numeric rating scale (NRS), characteristic pain intensity (CPI) and pain severity score (PSS)

Subgroup analyses were carried out for both primary outcomes based on the use of Research Diagnostic Criteria (RDC/TMD) and TMDs origin (muscular, articular or both). Separate analyses were performed according to treatment group, i.e. non-occluding splint and occlusal oral appliances use.

Secondary outcomes were: maximum mouth opening (mm), muscle tenderness reduction, temporomandibular joint (TMJ) lateral and posterior tenderness reduction and depression (Modified Symptom Checklist 90-revised (SCL-90R) instruments from Axis II RDC/TMD and Center for Epidemiologic Studies-Depression scale (CES-D)).

### Data collection and analysis

#### Selection of studies

All relevant studies were assessed and evaluated by two reviewers using the previously defined criteria. Two independent reviewers (JKP and SD) read and analyzed titles and abstracts. Disagreements were solved by a third author (BM).

#### Data extraction and management

Data extraction was conducted by two independent authors (JKP and SD) and supervised by third author (BM). Authors agreement were appraised using Kappa statistics. Agreement was accomplished (Kappa: 0.75–0.78). The reviewers used standardized predefined form, thus ensuring straight forward manner in extracting data from each eligible study for study methods (randomization procedure, method of allocation, blindness, design and duration), participants (sample size, age, gender, diagnostic criteria, origin of TMDs, TMDs symptoms duration, pain intensity on VAS before therapy, length of SS use during the day and the presence of dropouts), the type of interventions and the primary and secondary outcomes that were observed.

#### Assessment of risk of bias in included studies

All included studies were evaluated in consent with the Cochrane Handbook for Systematic Reviews of Interventions Version 5.1.0 [30]. The risk of bias was assessed by two reviewers (JKP and NM) using the Cochrane Collaboration's tool with response options of "Low risk" "Unclear risk" "High risk" for following criteria: sequence generation, allocation concealment, blinding, incomplete outcome date, reporting bias and other biases. Inconsistencies between reviewers were resolved by consensus and discussion.

#### Measures of treatment effect

For dichotomous outcome, the estimate of the effect of interventions was expressed as odds ratio (OR) together with 95% confidence intervals (95% CI). Standardized mean difference (SMD) and 95% CI were used for continuous outcomes.

#### Dealing with missing data

When necessary, the authors from primary studies were asked for information about missing data. When there was no response, this was addressed in the assessment of risk of bias.

#### Assessment of heterogeneity

Cochran *q* test for heterogeneity was performed for each meta-analysis (Chi square statistic). In addition, extent of inconsistency of treatment effects across trials were measured (I^2^ statistic; the percentage of total variance across studies that is due to heterogeneity rather than chance).

#### Assessment of reporting biases

Publication bias was appraised using the symmetry of funnel plots.

#### Methodological quality assessment

Jadad score was used to evaluate the quality of the studies [31].

#### Strength of evidence

Summary of Finding (SoF) table was created to assess the strength of the evidence in results using GRADEpro (Version 2016, McMaster University, 2014).

#### Data synthesis

Meta-analysis was performed in accordance with the rules adopted by the Cochrane Collaboration—Cochrane Reviewer's Handbook [32]. We used random effects method to summarize outcomes of interest. For each endpoint, a separate forest plot was described demonstrating OR or SMD as a box and 95% CI as whiskers on both sides of the box with the size of the box corresponding to the weight of each trial. Summary effect measures (OR or SMD) are also calculated and presented. All statistical analyses were performed using The Cochrane Collaboration Review Manager (RevMan), version 5.3 (The Cochrane Collaboration, Oxford, England). Univariate meta-regression model was performed for OR and MD as dependent variables. Meta-regression was conducted using R language and environment for statistical computing (R package version 3.8–0.; metafore package).

## Results

### Studies characteristics

**Fig 1:**
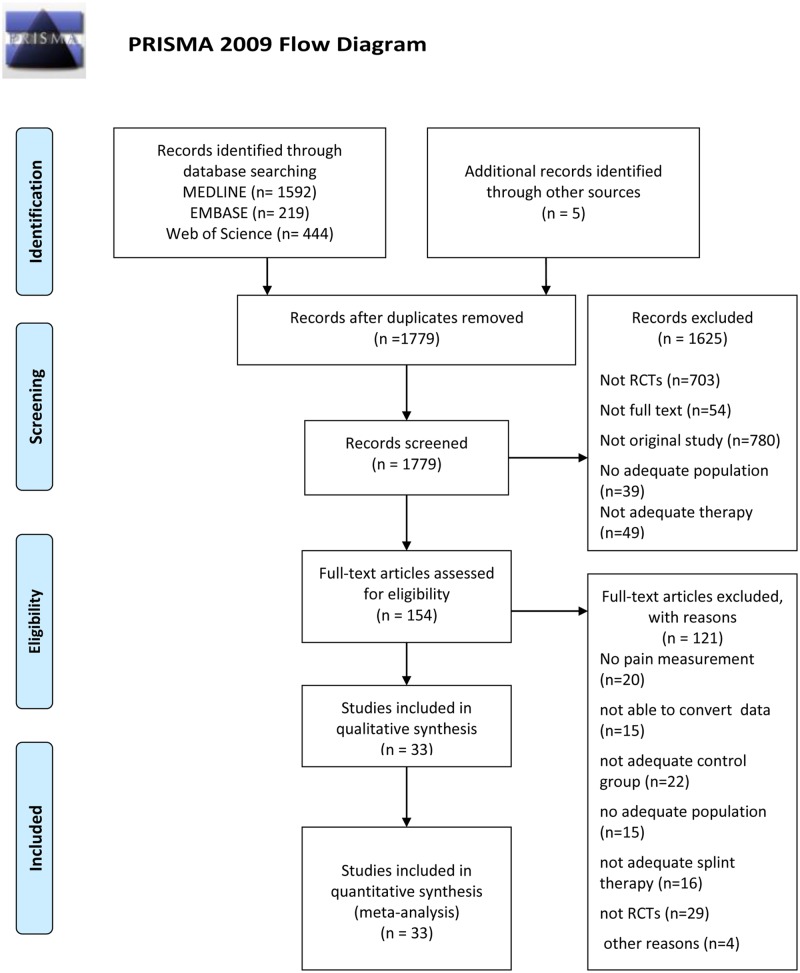
Flow chart diagram. *From*: Moher D, Liberati A, Tetzlaff J, Altman DG, The PRISMA Group (2009). *P*referred *R*eporting *I*tems for *S*ystematic Reviews and *M*eta-*A*nalyses: The PRISMA Statement. PLoS Med 6(7): e1000097. doi: 10.1371/journal.pmed1000097. **For more information, visit** www.prisma-statement.org.

Thirty three eligible RCTs were included in meta-analysis. The study selection process is presented in Fig 1. Ten studies had control group with non-occluding splint [17, 19, 33–40]. Nine studies compared SS with occlusal appliances [16, 18, 20, 41–46]. Five studies had control group with physical therapy [47–51]. Four studies compared SS vs. behavioral treatment [52–55]. Three studies included minimal treatment (exercise and counseling) [21, 46, 56], and one study used counseling as control group [45]. Seven studies had no treatment as control group [44, 50, 51, 54, 57–59]. Six studies had two control groups [44–46, 50, 51, 54]. Six studies were followed for short and long term outcomes [16, 46, 52–55]. The total number of included participants was 1779, which were divided to 848 in SS group and 931 patients in control group. Overviews of included studies (S1 Table) and references of excluded studies (S2 File) for this review are shown in supporting material.

#### Methodological quality assessment

The quality of the studies was assessed using the Jadad score. The overall mean score was 3.13. Five studies showed high methodological quality and achieved 5 points (S1 Table).

#### Risk of bias

**Fig 2:**
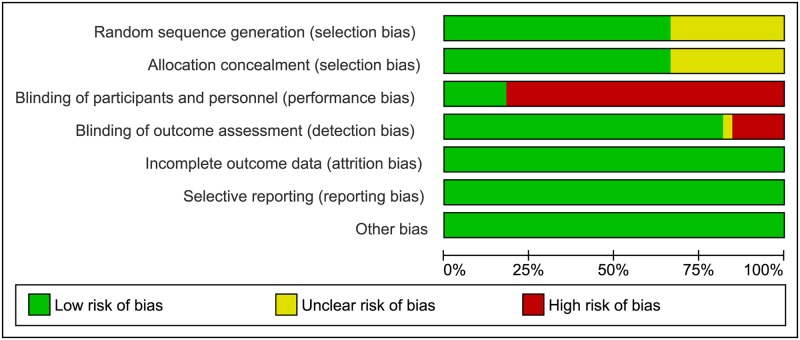
Risk of bias graph: Review authors' judgments about each risk of bias item presented as percentages across all included studies.

Fig 2 shows risk of bias as percentages across all included studies. More than 30% had unclear risk for selection bias (random sequence generation and allocation concealment). Around 80% were high risk for performance bias. 21 RCTs were single blinded for personnel and assessed as high risk, while six studies weren't blind nor for participants nor personnel. Six studies were double blinded RCTs. In blinding of outcome assessment (detection bias) 15% of studies had high risk. The funnel plots suggest no publication bias for pain reduction and pain intensity at short term (S1 and S2 Figs).

#### Strength of evidence

Gathered information regarding quality of included studies is presented in SoF table (S2 Table). The evaluation of results for two primary outcomes, followed both in short and long term, showed low quality. Assessment of secondary outcomes in short term (muscle tenderness reduction, TMJ lateral and posterior tenderness reduction, maximum mouth opening and depression) presented moderate quality.

### Data synthesis

#### Primary outcomes in short term follow-up

**Fig 3:**
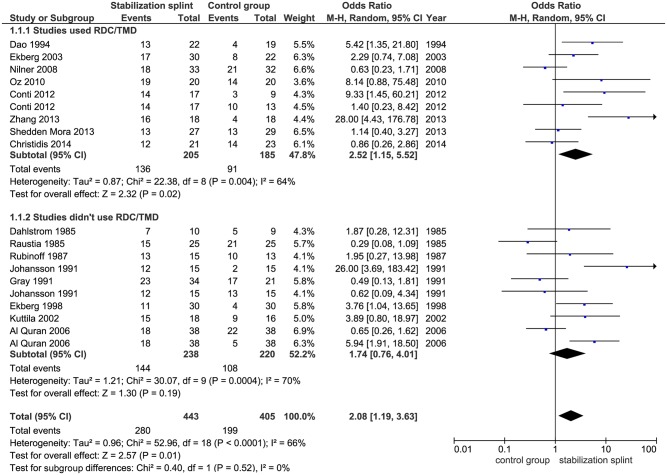
Forest plot comparison: Stabilization splint vs. Control group. Pain reduction according to RDC/TMD at short term.

**Fig 4:**
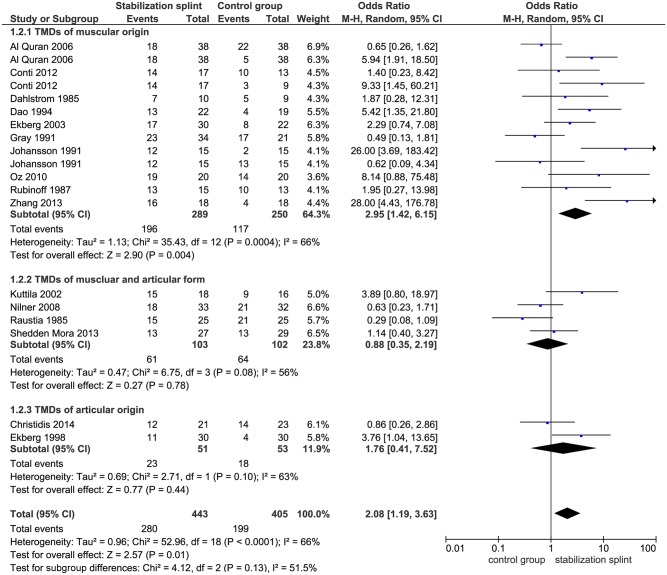
Forest plot comparison: Stabilization splint vs. Control group. Pain reduction according to TMDs origin at short term.

**Fig 5:**
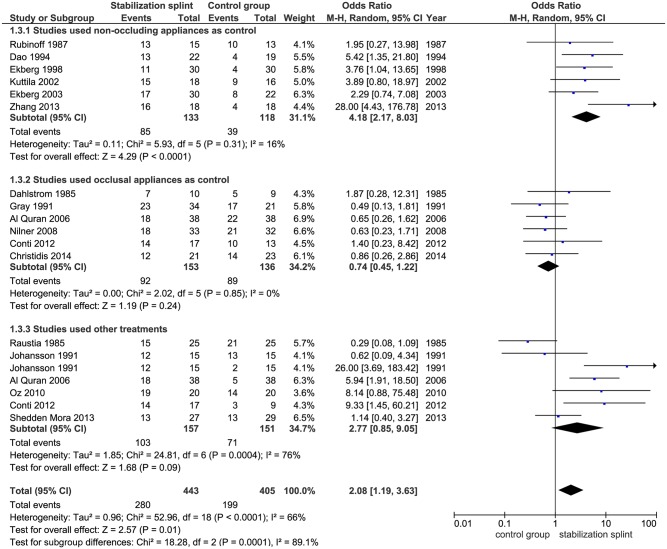
Forest plot comparison: Stabilization splint vs. Control group. Pain reduction according to individual treatments at short term.

Pain reduction. In short term, SS presented positive overall effect on pain reduction (OR 2.08; 95% CI [1.19, 3.63]; p = 0.01; I^2^ = 66%), based on 16 identified studies evaluating this outcome, with a total of 848 participants. Subgroup analysis revealed that in studies using RDC/TMD, SS had positive effect on pain reduction (OR 2.52; 95% CI [1.15, 5.52]; p = 0.02; I^2^ = 64%), but not for studies that didn’t use RDC/TMD (OR 1.74; 95% CI [0.76, 4.01]; p = 0.19; I^2^ = 70%) (Fig 3). The pooled results from 10 studies showed significant difference between SS and control group in patients with TMDs of muscular origin (OR 2.95; 95% CI [1.42, 6.15]; p = 0.004; I^2^ = 66%) (Fig 4). Six studies evaluated SS in comparison to non-occluding splint, and pointed out significant effect of SS (OR 4.18; 95% CI [2.17, 8.03]; p<0.0001; I^2^ = 16%), while comparison of SS versus occlusal oral appliances showed no difference between groups (based on 6 included studies) (OR 0.74; 95% CI [0.45, 1.22]; p = 0.24; I^2^ = 0%) (Fig 5).

**Fig 6:**
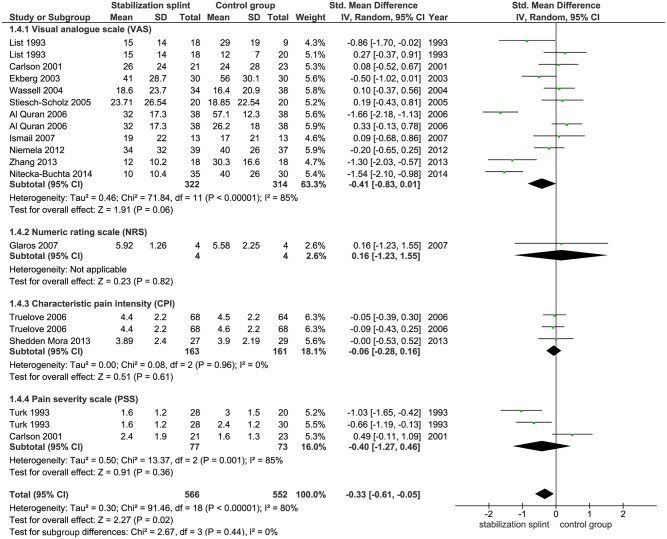
Forest plot comparison: Stabilization splint vs. Control group. Pain intensity according to numeric scales at short term.

**Fig 7:**
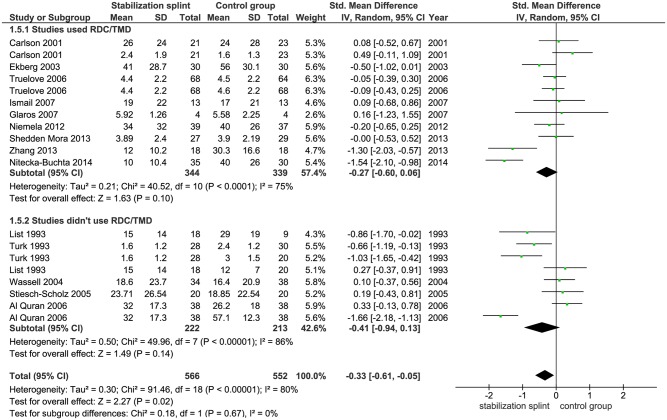
Forest plot comparison: Stabilization splint vs. Control group. Pain intensity according to RDC/TMD at short term.

**Fig 8:**
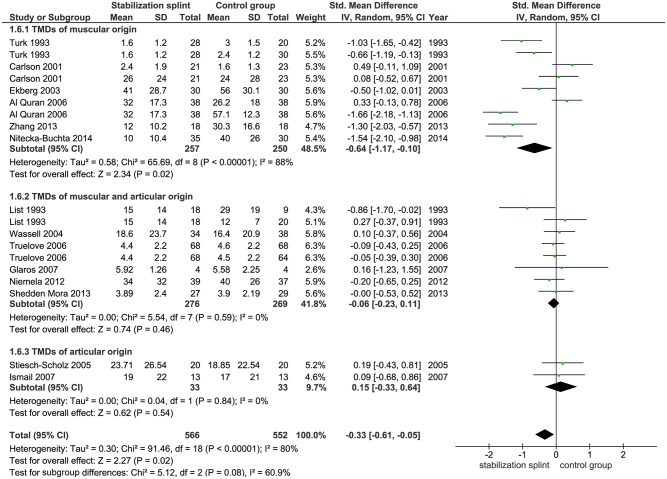
Forest plot comparison: Stabilization splint vs. Control group. Pain intensity according to TMDs origin at short term.

Pain intensity. Fifteen identified studies evaluated pain intensity with total of 1118 participants. Ten studies used VAS, one study used NRS, while two studies each used CPI and PSS, respectively. Meta-analysis demonstrated statistically significant difference between groups in favor of the SS (SMD -0.33; 95% CI [-0.61, -0.05]; p = 0.02; I^2^ = 80%) (Fig 6). Subgroup analyses showed no difference in SS effect according to the use of RDC/TMD criteria (SMD -0.27; 95% CI [-0.60, 0.06]; p = 0.10; I^2^ = 75% for studies that used RDC/TMD; SMD -0.41; 95% CI [-0.94, 0.13]; p = 0.14; I^2^ = 86% for studies that didn’t use RDC/TMD) (Fig 7). The pooled results from 6 studies that evaluated TMDs of muscular origin showed significant difference between groups (SMD -0.64; 95% CI [-1.17,-0.10]; p = 0.02, I^2^ = 88%) in favor of the SS (Fig 8).

#### Primary outcomes in long term follow-up

**Fig 9:**
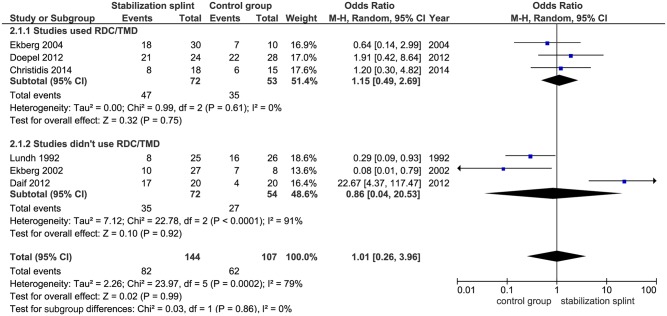
Forest plot comparison: Stabilization splint vs. Control group. Pain reduction at long term.

Pain reduction. Long term results showed no difference in pain reduction between groups, based on six observed studies with total of 251 participants (OR 1.01; 95% CI [0.26, 3.96]; p = 0.99; I^2^ = 79%). Subgroup analyses presented the same effects both in studies that used RDC/TMD (OR 1.15; 95% CI [0.49, 2.69]; p = 0.75; I^2^ = 0%) and that didn’t use RDC/TMD (OR 0.86; 95% CI [0.04, 20.53]; p = 0.92; I^2^ = 91%) (Fig 9).

**Fig 10:**
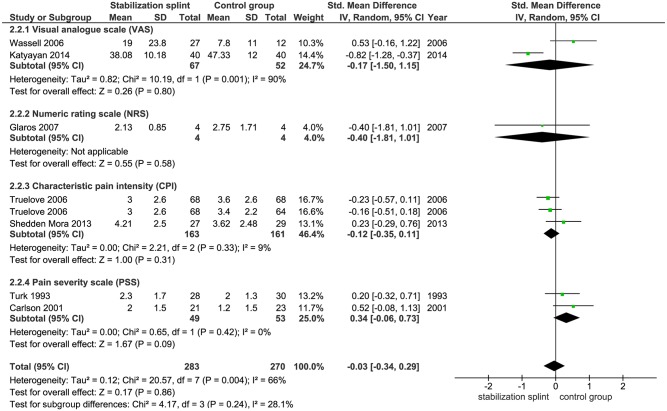
Forest plot comparison: Stabilization splint vs. Control group. Pain intensity on according to numeric scales at long term.

**Fig 11:**
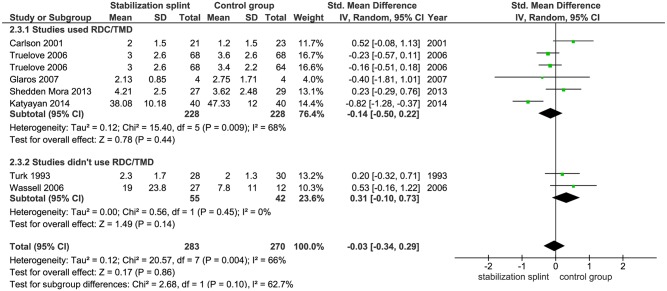
Forest plot comparison: Stabilization splint vs. Control group. Pain intensity according to RDC/TMD at long term.

Pain intensity. Seven studies with 553 participants followed the long-term effects of SS therapy on pain intensity, out of which two studies used VAS, CPI and PSS, while one study used NRS. There was no statistically significant difference between SS and control group (SMD -0.03 [-0.34, 0.29]; p = 0.86; I^2^ = 66%) (Fig 10). Subgroup analyses showed the same effects both in studies that used RDC/TMD (SMD -0.14 [-0.50, 0.22]; p = 0.44; I^2^ = 68%) and that didn’t use RDC/TMD (SMD 0.31 [-0.10, 0.73]; p = 0.14; I^2^ = 0%) (Fig 11).

#### Secondary outcomes in short and long term follow-up

Muscle tenderness reduction. Significant decrease of number of patients with muscle tenderness was found for SS group in short term, based on four studies with 194 participants (OR 1.97; 95% CI [1.05, 3.68]; p = 0.03; I^2^ = 0%) (S3 Fig).

TMJ lateral and posterior tenderness reduction. Results based on five studies with 270 participants showed no important difference between compared groups in short term (OR 1.05; 95% CI [0.53, 2.08]; p = 0.89; I^2^ = 15%) (S4 Fig).

Maximum mouth opening. Assessing the results from seven studies with 298 participants, SS showed significant effect compared to control group (SMD -0.30; 95% CI [-0.59, -0.01]; p = 0.04; I^2^ = 33%) (S5 Fig). In evaluating the long term effect, 3 included studies showed no significant difference between SS intervention and control group (SMD -0.12; 95% CI [-0.44, 0.21]; p = 0.47; I^2^ = 0%) (S6 Fig).

Depression. In short term, five studies with 290 participants assessed the effect of SS on depression. Two studies used CES-D scale, while 3 studies used SCL-90R scale. Results showed no important difference between compared groups (SMD -0.09; 95% CI [-0.44, 0.27]; p = 0.63; I^2^ = 56%) (S7 Fig). Long term results based on five studies with 243 participants (2 studies used CES-D scale and three studies used SCL-90R scale) pointed out statistically significant difference between SS and control (SMD -0.30; 95% CI [0.05, 0.56]; p = 0.02; I^2^ = 0%) in favor of the control group (S8 Fig).

#### Meta-regression

The influence of independent factors on the effect size of SS for pain reduction and pain intensity was assessed by meta-regression analysis in short time follow-up. Univariate meta-regression model was performed for OR and SMD as dependent variables. The following factors were examined: age, sex, therapy duration, TMDs symptoms duration, pain intensity on VAS before therapy, length of SS use during the day and the presence of dropouts. After analysis of factors for both primary outcomes, length of use SS during the day was singled out as significant factor for pain reduction (meta-regression coefficient was 1.73 (SE 0.69; p = 0.011)). These results presented that the effect of SS was better in patients in which splint was present for 24 hours than in patients who used it only during the night (S3 Table).

## Discussion

Over the years, SS became the most frequent therapeutic choice for the treatment of TMDs, and the most evaluated one [60]. This meta-analysis observed RCTs for short and long term effects of SS compared to control group, and further investigated the role of validated diagnostic system in establishing therapy effect, while determining adequate course of treatment for particular origin of TMDs. Effect of SS was determined by the means of pain assessment, measured as the presence of pain reduction and/or estimated pain intensity. Pain reduction was defined as an improvement or reduction in signs and symptoms at the end of the treatment, while, numeric scales (such as VAS, NRS, CPI and PSS) are used for determining the pain intensity. Two of these numeric scales (VAS and NRS) represent pain intensity at the end of therapy, while CPI and PSS show levels of multiply measured pain intensity over the time. Using SMD in data synthesis, we were able to compare data gathered with these four different scales. Besides the pain assessment, the effect of SS on maximum mouth opening, muscle and TMJ tenderness and depression was also evaluated in order to gain additional insight in the overall success of SS therapy.

Regarding the pain reduction in short term evaluation (follow up less than 3 months), 16 studies were included, out of which 8 used RDC/TMD and 8 studies didn’t use these criteria. The overall effect presented significantly better results for SS and results of subgroup analysis revealed the same effect in studies that used RDC/TMD as validated diagnostic system for defining TMDs. Further, pooled results of meta-analysis based on 15 studies measuring pain intensity in short term were also in favor of SS.

A critical point was made by reviewers of different studies suggesting that patients with muscle problems should be separated from those with TMJ problems in future research [7, 61]. However, the most of published studies included all possible patient populations, sorting them afterwards to observe weather some of the displayed symptoms were affected by the given therapy [62]. Dealing with this issue, we conducted a seperate subgroup analysis according to TMDs origin to deeper inspect the effect of SS in different patient population. A significant difference in favor of SS for both primary outcomes was demonstrated in studies with TMDs of muscular origin.

In order to further investigate SS efficacy we carried out meta-analysis using different individual therapeutic modalities as a control group. First, comparison with the non-occluding splint use (type of placebo splint) demonstrated significant difference between those in short term. SS showed better effect in pooled results of all studies examining pain reduction, which is in accordance with the results of Fricton et al. [24], but in contrast to Al Ani et al. that presented no difference between the groups [25]. To the best of our knowledge, this is the first meta-analysis that compared SS with other oral occlusal appliances for pain reduction, which presented no difference between the therapy groups. This indicates that patients with TMDs besides SS may benefit from other occlusal appliances in reducing symptoms of TMDs. This conclusion may support the further research of the effect of occlusal therapy in more well-designed RCTs in order to gain clearer results of possible effects of SS as well as other occlusal therapies in treatment of TMDs. Meta-regression performed to determine the influence of different independent factors on SS efficacy, identify only the continuous use of SS (24 hours a day) as contributing factor in reducing the symptoms of TMDs. Based on the results, wearing the splint 24 hours per day stabilizes jaw position resulting in occlusal stability.

In an evaluation of the long term effects of therapy, our results demonstrated that there were no significant differences between SS and control group. In total, 6 studies followed the effect for pain reduction, while 7 studies followed pain intensity. Subgroup analyses presented the same effects both in studies that used RDC/TMD and that didn’t use RDC/TMD. Just to clarify, all studies didn’t use the same time frame for tracking the effect of SS, nor they used same timetable of wearing the splint during the day. For pain reduction outcome, 2 studies followed patients for 6 months and in that time patients wore splint during the night [20, 58]. Four studies followed participants for 1 year, where in one study patients wore splint during the course of therapy only by night [57], 2 studies where approximately 50% of patients wore splint during the night [16, 36], while in one study 47% of participants used splint several times in a week [19]. Furthermore, for pain intensity, 4 studies followed the effect of therapy over 6 months. One study estimated wearing a splint at all times during the therapy [52], two studies did not wear splint upon completion of short-term therapy [53, 54], while in one study participants continued to wear splint by night [21]. 3 studies followed the SS effect for a year where in one study patients wore splint only during short term treatment [55], while in two studies splint was worn at night by 50% of participants [40, 46]. Taking into account effects for both short and long term therapies, the overall results showed that SS is more effective in treating patients with TMDs in short term period, while the long term effect of SS is equalized with other therapeutic modalities.

Observing the secondary outcomes for short term therapies, results demonstrated benefit for SS group in terms of muscle tenderness reduction and improved maximum mouth opening. Results for TMJ lateral and posterior tenderness reduction and depression presented no important difference between observed groups. In long term follow up of SS effect, similar results were obtained between compared groups for maximum mouth opening, while significant difference was found for depression in favor of the control group. Splint also increases vertical dimension of occlusion which relaxes jaw muscles. Our meta-analysis confirmed, evaluating pain outcomes and through reduction of muscle tenderness and maximum mouth opening, that patients with myogenous TMDs may have significant benefit from SS.

### Quality assessment

Jadad score used in assessing the methodological quality of RCTs included in meta-analysis showed moderate quality of included RCTs studies. Main cause for this estimation was the lack of double blinded design of RCTs. Furthermore, assessment of risk of bias also presented flaws of blinding process. More than 80% of studies were high risk for performance bias, while 15% of studies were high risk for blinding of outcome assessment (detection bias). Publication bias, as form of selection bias showed no important asymmetry in none of the observed outcomes (pain reduction and pain intensity) in the funnel plots.

### Strength of evidence

Evaluation of primary outcomes using SoF table presented low quality of evidence for primary outcomes in both short and long term period. The first reason for this is the presence of risk of bias which was estimated as serious due to lack of blinding process, unclear allocation concealment and random sequence generation. The second reason for low quality of evidence is moderate heterogeneity. Assessment of secondary outcomes shows moderate quality of evidence in short term (S2 Table).

### Comparison with other reviews

Some of the most prominent systematic reviews presented different results for the effect of SS in relation to other types of therapy. There are a number of limitations that challenge the interpretation of the results of these meta-analyses. Careful attention to these caveats is not only the key for critical evaluation of the literature, but it also has implications for treatment of patients, and for the development and implementation of practice guidelines. There are several systematic reviews and meta-analysis that analyzed effect of SS with other therapies separately, while other combined SS with other therapeutic modalities [1, 12, 24–26]. Few important issues are distinguishing our meta-analysis from others. First of all, in order to evaluate more accurately the effect of SS, we used two primary outcomes for pain assessment followed by several secondary outcomes. This provided more valid data of SS efficacy. Also, we wanted to investigate the importance of using validated diagnostic system and its influence on overall effect of SS. Further, dividing studies according to TMDs origin enabled deeper insight in which population should benefit more from wearing SS. Fricton et al. in their systematic review demonstrated the advantage of SS versus non-occluding splint and minimal treatment for pain reduction [24]. A systematic review of Al-Ani et al. showed no significant difference in the therapeutic effect of SS versus non-occluding splints for pain outcome. They also presented little evidence for SS compared with other forms of therapy [25]. Furthermore, there are systematic reviews that evaluated effects of different types of appliances without separating SS exclusively. Ebrahim et al. in their meta-analysis found significant results comparing the effect of different types of splints for pain reduction with minimal treatment or no treatment, without separating the studies on short term and long term [12]. Results from systematic review of Aggarwal et al. showed comparison of psychosocial therapy versus usual treatment and presented significantly better results for usual treatment (occlusal splint, exercise and drugs) versus CBT in short term for pain outcome. In long term, CBT has an advantage over the usual treatment [26]. According to a recent review on this topic, study of Roldan-Barazza et al. showed that psychosocial therapy achieves better effects for pain outcome for long term, while in short term there is no difference [1].

Our results confirmed that the effect of SS for pain outcome in short term is significantly better than control group, while for long term studies the effect fades away. Small number of studies addressed this issue concerning the long term effect of SS and continued following patients habits of wearing splint. All of them presented that approximately 50–60% of patients continued wearing splint during the night [16, 36], while others ceased wearing the splint completely [53, 54]. Roldan-Baraza et al. showed that the usual treatment had greater efficacy for mouth opening, which was also confirmed by our results [1]. Ebrahim et al. in his study also analyzes the effect of therapy on depression and demonstrates that there is no significant difference between compared groups [12], while Aggarwal et al. presented statistically significant difference between psychosocial treatment and usual treatment in long term studies [26]. Our results indicate that there is no significant difference between the SS and other therapies for depression in the short term, however results are in favor of the control group for long term studies. These results showed that SS can affect the psyche of patients prone to depression in short term, but in long term other therapies are better for this particular population.

### Advantages and limitations of the study

The advantage of this meta-analysis is the isolation of SS effect and its comparison to other therapies altogether, in short and long term period. Subgroup analyses of outcomes based on RDC/TMD are another advantage of the study. Separation of included studies whether they used RDC/TMD for pain assessment or not, provided more valid results of our analysis and presented the importance of these criteria in estimation of observed outcomes. This was confirmed using CPI for pain outcome and SCL-90R for depression. Also, subgroup analyses by diagnosis and different control groups gave an additional insight into the SS effect in different subpopulations and treatment strategies. Finally, meta-regression provided better insight into the factors influencing SS efficacy.

Limitation of this meta-analysis is the presence of heterogeneity between pooled studies for primary outcomes. Comparison of SS with the control group leads to a moderate or high heterogeneity. This resulted in low quality of evidence for primary outcomes in both short and long term period. The lack of data in all studies was a limitation of meta-regression. Low quality of some studies may influence the overall results, and at the same time overconfidence may occur in pain assessment causing more positive results than they actually are.

### Recommendations

The overall results of this meta-analysis pointed out that in future studies investigators should use clearly defined criteria for assessing efficacy of therapeutic modalities for treating TMDs. It is necessary to use the appropriate study design, i.e. RCTs to determine the efficacy of therapy under assessment, which should be conducted according to the relevant reporting guidelines (CONSORT Statement) [63]. The importance of randomization, allocation concealment and blinding process in these studies should be clearly defined. Beside the well-conducted RCTs, investigators should pay attention not only on short term effects but also insist in pursuing long term therapy effects. Studied population should be based on standardized criteria for diagnosing of TMDs (RDC/TMD or DC/TMD). The sample size should be determined a priori for each study in order to be able to identify the true effect of the treatment. Lastly, appropriate scale measurement for pain outcome should be established using characteristic pain intensity from RDC/TMD criteria and used in straight-forward manner for the assessment of therapy effect. In addition, presented results should provide all the information needed for secondary analysis.

## Conclusion

SS may have a significant role in treating TMDs in short term, while its effect is equalized with other therapeutic modalities in long term follow up. Further studies based on appropriate use of standardized criteria for patient recruitment and outcomes under assessment are needed to better define SS treatment modalities that may influence its effect persistence in long term.

## Supporting information

S1 Checklist Prisma 2009 checklist. (DOC)

S1 Fig Funnel plot comparison: Stabilization splint vs. Control group. Pain reduction according to RDC/TMD at short term.
(TIF)

S2 Fig Funnel plot comparison: Stabilization splint vs. Control group. Pain intensity according to RDC/TMD at short term.
(TIF)

S3 Fig Funnel plot comparison: Stabilization splint vs. Control group. Muscle tenderness reduction at short term.
(TIF)

S4 Fig Funnel plot comparison: Stabilization splint vs. Control group. TMJ lateral and posterior tenderness reduction at short term.
(TIF)

S5 Fig Funnel plot comparison: Stabilization splint vs. Control group. Maximum mouth opening at short term.
(TIF)

S6 Fig Funnel plot comparison: Stabilization splint vs. Control group. Maximum mouth opening at long term.
(TIF)

S7 Fig Funnel plot comparison: Stabilization splint vs. Control group. Depression at short term.
(TIF)

S8 Fig Funnel plot comparison: Stabilization splint vs. Control group. Depression at long term.
(TIF)

S1 File Search strategy for MEDLINE (PubMed). (DOCX)

S2 File References of excluded studies from this review. (DOCX)

S1 Table Descriptive characteristics of included studies. (DOC)

S2 Table Grade rating system. (PDF)

S3 Table Univariate meta-regression model. (DOC)
