# Supplementary figures and images for: Occlusal stabilization splint for patients with temporomandibular disorders: Meta-analysis of short and long term effects
Source: PLoS One. 2017 Feb 6;12(2):e0171296. doi: 10.1371/journal.pone.0171296 (PMC5293221; doi:10.1371/journal.pone.0171296)

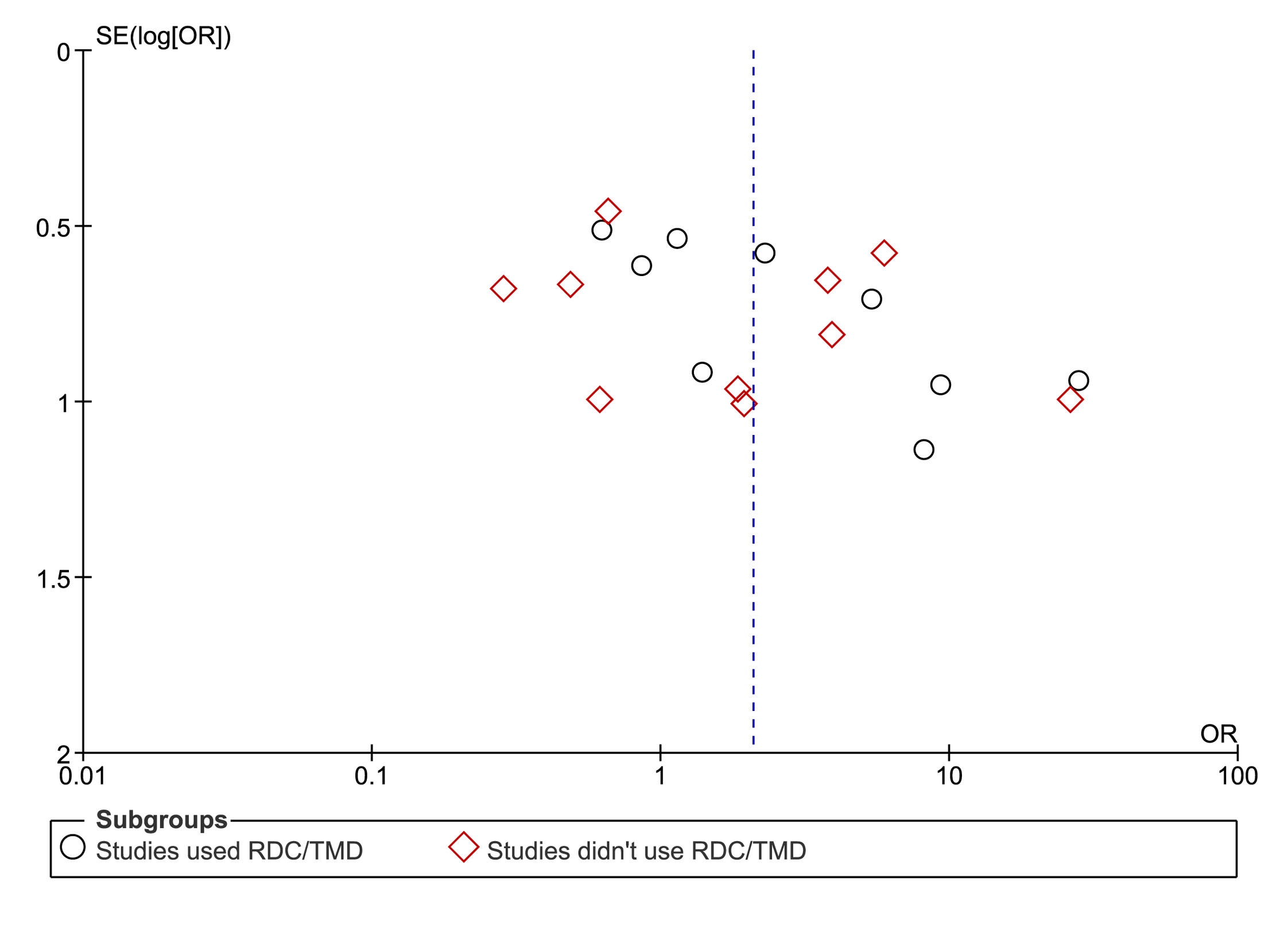

Supplement: S1 Fig — Pain reduction according to RDC/TMD at short term. (TIF) [file pone.0171296.s002.tif]

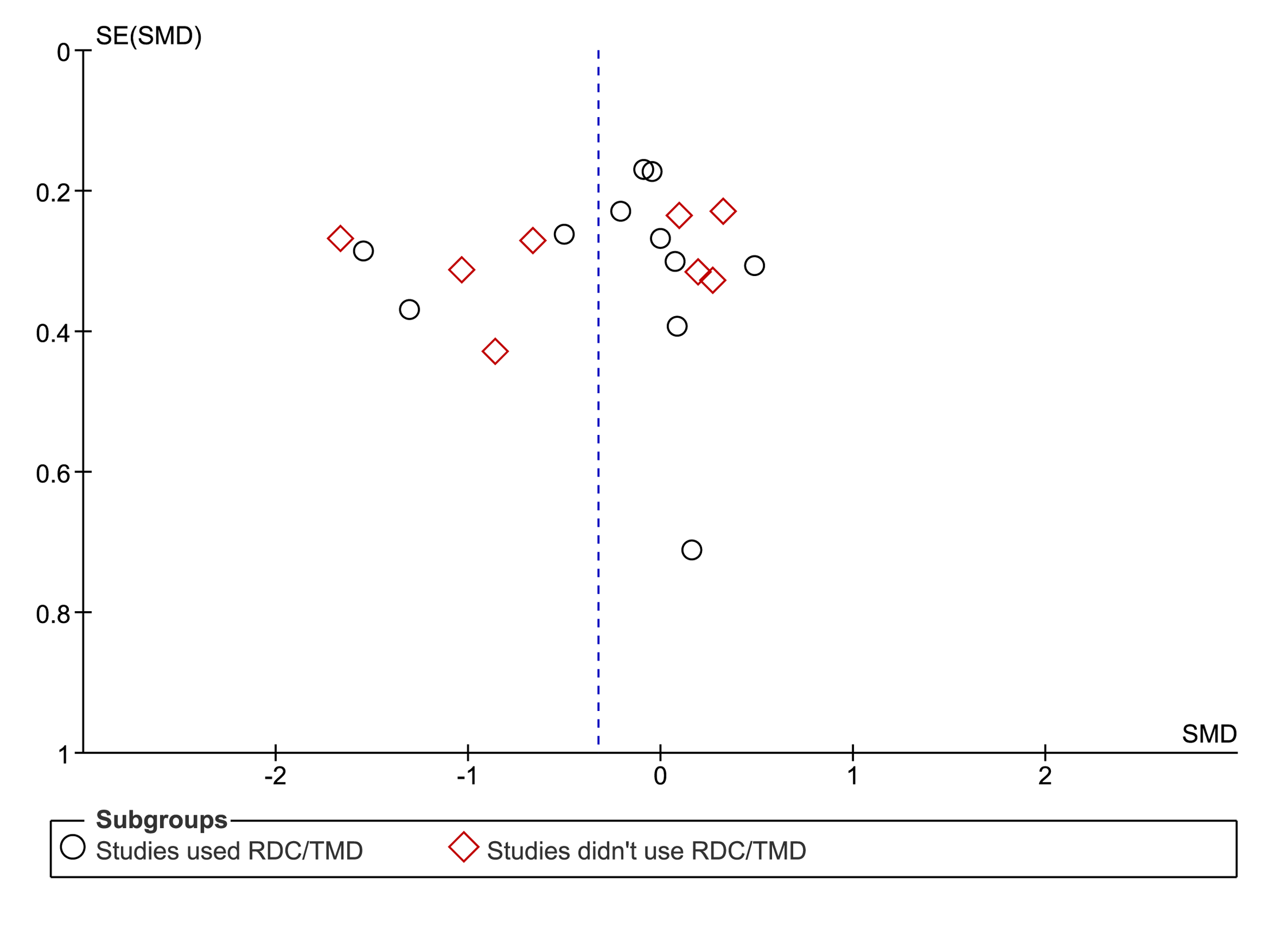

Supplement: S2 Fig — Pain intensity according to RDC/TMD at short term. (TIF) [file pone.0171296.s003.tif]

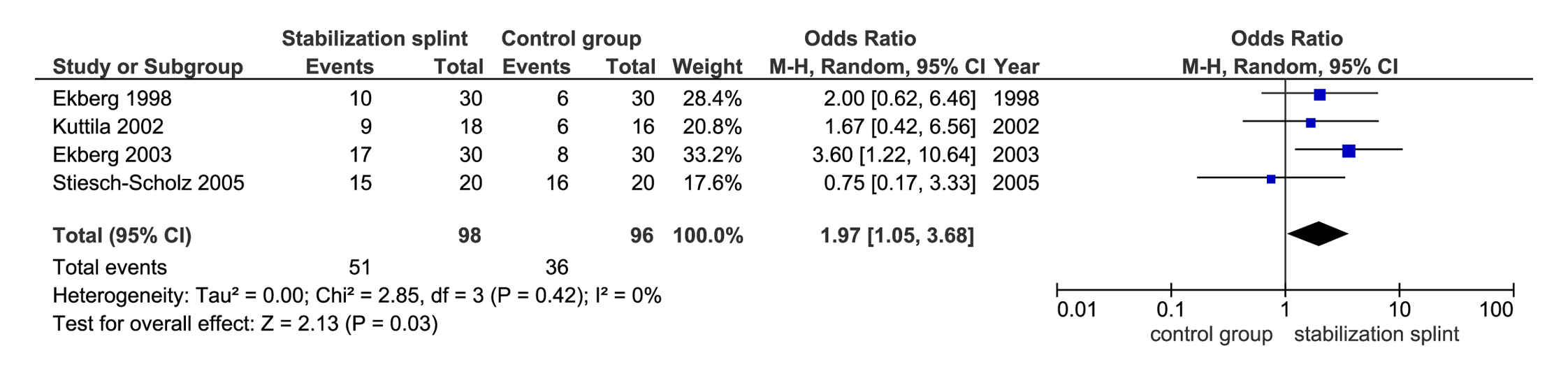

Supplement: S3 Fig — Muscle tenderness reduction at short term. (TIF) [file pone.0171296.s004.tif]

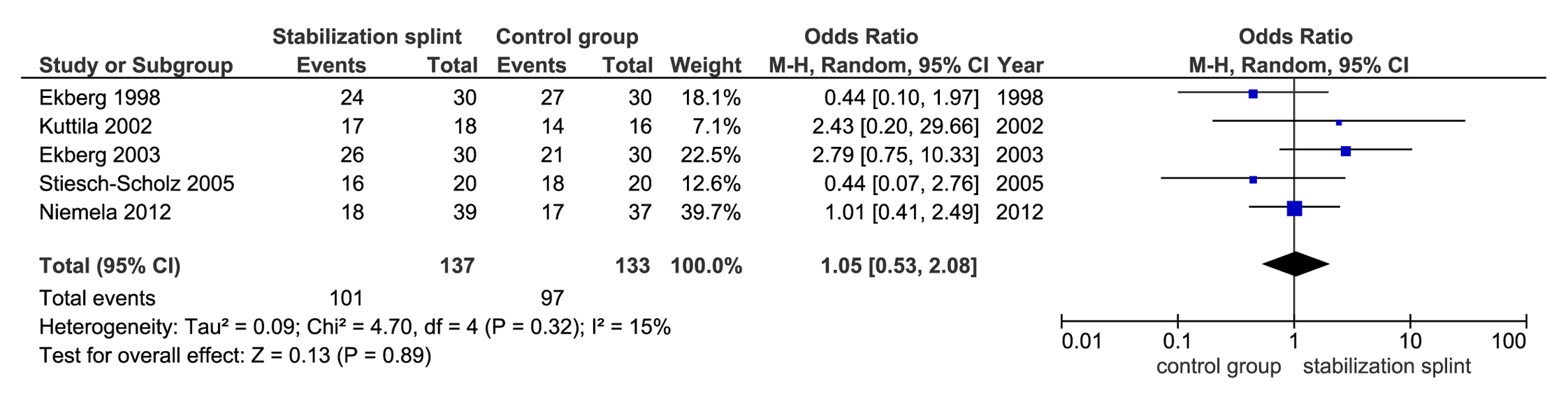

Supplement: S4 Fig — TMJ lateral and posterior tenderness reduction at short term. (TIF) [file pone.0171296.s005.tif]

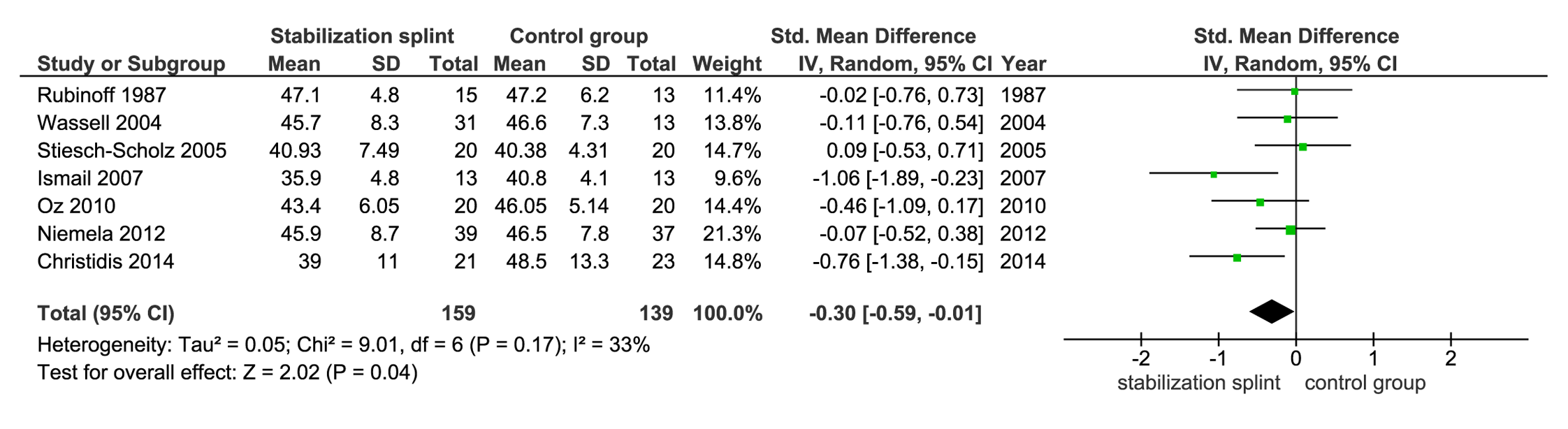

Supplement: S5 Fig — Maximum mouth opening at short term. (TIF) [file pone.0171296.s006.tif]

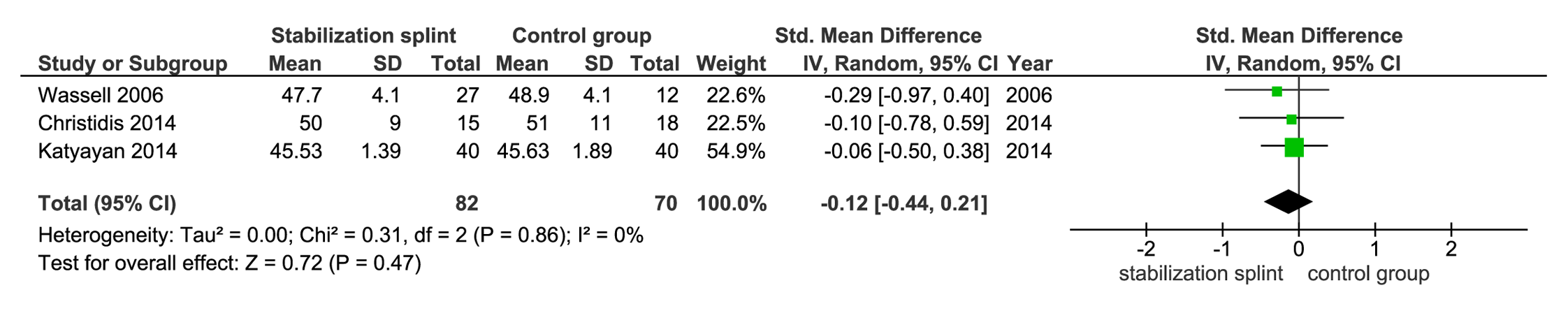

Supplement: S6 Fig — Maximum mouth opening at long term. (TIF) [file pone.0171296.s007.tif]

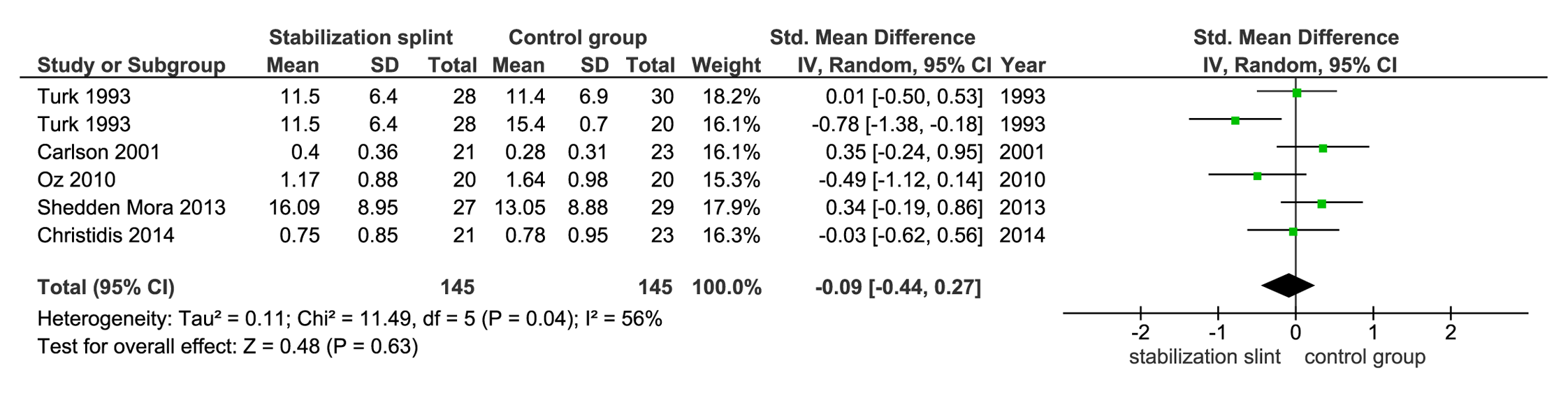

Supplement: S7 Fig — Depression at short term. (TIF) [file pone.0171296.s008.tif]

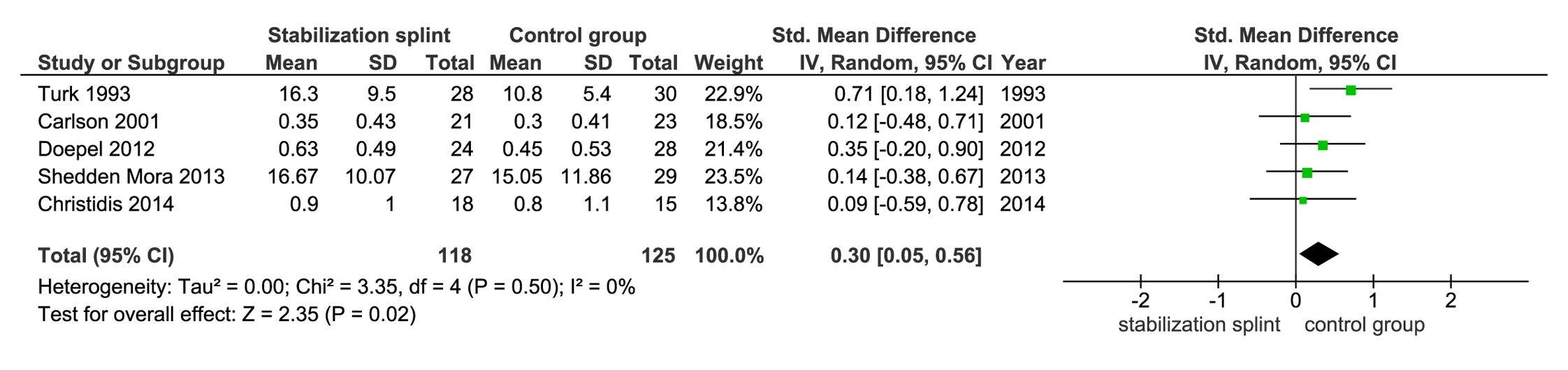

Supplement: S8 Fig — Depression at long term. (TIF) [file pone.0171296.s009.tif]
