# Supplementary material for: Occlusal stabilization splint for patients with temporomandibular disorders: Meta-analysis of short and long term effects
Source: PLoS One. 2017 Feb 6;12(2):e0171296. doi: 10.1371/journal.pone.0171296 (PMC5293221; doi:10.1371/journal.pone.0171296)
Supplement: S1 File — (DOCX) [file pone.0171296.s010.docx]

**S1 File.** Search strategy for MEDLINE (PubMed)

Controlled vocabulary is given in upper case type and free text terms in lower case

1. TEMPOROMANDIBULAR JOINT DISORDERS

2. temporomandibular disorders

3. CRANIOMANDIBULAR DISORDERS

4. myofacial pain

5. myofascial pain

6. orofacial pain

7. FACIAL PAIN

8. HEADACHE

9. OR/ 1-8

10. stabilization splint

11. OCCLUSAL SPLINTS

12. occlusal appliance

13. splint therapy

14. OR/10-13

15. 9 AND 14
