# Supplementary material for: Occlusal stabilization splint for patients with temporomandibular disorders: Meta-analysis of short and long term effects
Source: PLoS One. 2017 Feb 6;12(2):e0171296. doi: 10.1371/journal.pone.0171296 (PMC5293221; doi:10.1371/journal.pone.0171296)
Supplement: S2 File — (DOCX) [file pone.0171296.s011.docx]

**S2 File.** References of excluded studies from this review

**No pain measurement**

1. Anderson GC, Schulte JK, Goodkind RJ. Comparative study of two treatment methods for internal derangement of the temporomandibular joint. J Prosthet Dent. 1985;53(3):392-7. Epub 1985/03/01. PubMed PMID: 3857335.

2. Carlson N, Moline D, Huber L, Jacobson J. Comparison of muscle activity between conventional and neuromuscular splints. J Prosthet Dent. 1993;70(1):39-43. Epub 1993/07/01. PubMed PMID: 8366456.

3. Costa YM, Porporatti AL, Stuginski-Barbosa J, Bonjardim LR, Conti PC. Additional effect of occlusal splints on the improvement of psychological aspects in temporomandibular disorder subjects: A randomized controlled trial. Arch Oral Biol. 2015;60(5):738-44. Epub 2015/03/15. doi: 10.1016/j.archoralbio.2015.02.005. PubMed PMID: 25768709.

4. Costa YM, Porporatti AL, Stuginski-Barbosa J, Bonjardim LR, Specie JG, Conti PCR. Headache Attributed to Masticatory Myofascial Pain: Clinical Features and Management Outcomes. Journal of Oral & Facial Pain and Headache. 2015;29(4):323-30. PubMed PMID: WOS:000364609600002.

5. Doepel M, Nilner M, Ekberg E, Vahlberg T, le Bell Y. Headache: short- and long-term effectiveness of a prefabricated appliance compared to a stabilization appliance. Acta Odontologica Scandinavica. 2011;69(3):129-36. doi: 10.3109/00016357.2010.538719. PubMed PMID: WOS:000289561800001.

6. Doepel M, Soderling E, Ekberg EL, Nilner M, Le Bell Y. Salivary cortisol and IgA levels in patients with myofascial pain treated with occlusal appliances in the short term. J Oral Rehabil. 2009;36(3):210-6. Epub 2008/12/05. doi: 10.1111/j.1365-2842.2008.01923.x. PubMed PMID: 19054288.

7. Ekberg EC, Nilner M. Treatment outcome of short- and long-term appliance therapy in patients with TMD of myogenous origin and tension-type headache. Journal of Oral Rehabilitation. 2006;33(10):713-21. doi: 10.1111/j.1365-2842.2006.01659.x. PubMed PMID: WOS:000240028200001.

8. Ekberg E, Nilner M. The influence of stabilisation appliance therapy and other factors on the treatment outcome in patients with temporomandibular disorders of arthrogeneous origin. Swed Dent J. 1999;23(1):39-47. Epub 1999/06/17. PubMed PMID: 10371004.

9. Ekberg E, Sabet ME, Petersson A, Nilner M. Occlusal appliance therapy in a short-term perspective in patients with temporomandibular disorders correlated to condyle position. International Journal of Prosthodontics. 1998;11(3):263-8. PubMed PMID: WOS:000073867600010.

10. Ekberg E, Vallon D, Nilner M. Treatment outcome of headache after occlusal appliance therapy in a randomised controlled trial among patients with temporomandibular disorders of mainly arthrogenous origin. Swedish dental journal. 2002;26(3):115-24. PubMed PMID: 35532419.

11. Fayed MM, El-Mangoury NH, El-Bokle DN, Belal AI. Occlusal splint therapy and magnetic resonance imaging. World J Orthod. 2004;5(2):133-40. Epub 2004/12/24. PubMed PMID: 15615131.

12. Ficnar T, Middelberg C, Rademacher B, Hessling S, Koch R, Figgener L. Evaluation of the effectiveness of a semi-finished occlusal appliance - a randomized, controlled clinical trial. Head & Face Medicine. 2013;9. doi: 10.1186/1746-160x-9-5. PubMed PMID: WOS:000314997600001.

13. Gomes CA, Politti F, Andrade DV, de Sousa DF, Herpich CM, Dibai-Filho AV, et al. Effects of massage therapy and occlusal splint therapy on mandibular range of motion in individuals with temporomandibular disorder: a randomized clinical trial. J Manipulative Physiol Ther. 2014;37(3):164-9. Epub 2014/01/07. doi: 10.1016/j.jmpt.2013.12.007. PubMed PMID: 24387891.

14. Gray RJ, Quayle AA, Hall CA, Schofield MA. Physiotherapy in the treatment of temporomandibular joint disorders: a comparative study of four treatment methods. British dental journal. 1994;176(7):257-61. PubMed PMID: 24904224.

15. List T. Acupuncture in the treatment of patients with craniomandibular disorders. Comparative, longitudinal and methodological studies. Swedish dental journal. 1992;Supplement. 87:1-159. PubMed PMID: 23867341.

16. Lundh H, Westesson PL, Jisander S, Eriksson L. Disk-repositioning onlays in the treatment of temporomandibular joint disk displacement: comparison with a flat occlusal splint and with no treatment. Oral Surg Oral Med Oral Pathol. 1988;66(2):155-62. Epub 1988/08/01. PubMed PMID: 3174047.

17. Lundh H, Westesson PL, Kopp S, Tillstrom B. Anterior repositioning splint in the treatment of temporomandibular joints with reciprocal clicking: comparison with a flat occlusal splint and an untreated control group. Oral Surg Oral Med Oral Pathol. 1985;60(2):131-6. Epub 1985/08/01. PubMed PMID: 3862019.

18. Raustia AM, Pohjola RT. Acupuncture compared with stomatognathic treatment for TMJ dysfunction. Part III: Effect of treatment on mobility. J Prosthet Dent. 1986;56(5):616-23. Epub 1986/11/01. PubMed PMID: 3464742.

19. Raustia AM, Pohjola RT, Virtanen KK. Acupuncture compared with stomatognathic treatment for TMJ dysfunction. Part II: Components of the dysfunction index. J Prosthet Dent. 1986;55(3):372-6. Epub 1986/03/01. PubMed PMID: 3457169.

20. Schokker RP, Hansson TL, Ansink BJ. The result of treatment of the masticatory system of chronic headache patients. J Craniomandib Disord. 1990;4(2):126-30. Epub 1990/01/01. PubMed PMID: 2133472.

**Not able to convert data**

1. Alencar F, Becker A. Evaluation of different occlusal splints and counselling in the management of myofascial pain dysfunction. Journal of Oral Rehabilitation. 2009;36(2):79-85. doi: 10.1111/j.1365-2842.2008.01913.x. PubMed PMID: WOS:000262673900001.

2. Amin A, Meshramkar R, Lekha K. Comparative evaluation of clinical performance of different kind of occlusal splint in management of myofascial pain. J Indian Prosthodont Soc. 2016;16(2):176-81. Epub 2016/05/04. doi: 10.4103/0972-4052.176521. PubMed PMID: 27141168; PubMed Central PMCID: PMCPMC4837781.

3. Conti PC, dos Santos CN, Kogawa EM, de Castro Ferreira Conti AC, de Araujo Cdos R. The treatment of painful temporomandibular joint clicking with oral splints: a randomized clinical trial. J Am Dent Assoc. 2006;137(8):1108-14. Epub 2006/07/29. PubMed PMID: 16873326.

4. Crockett DJ, Foreman ME, Alden L, Blasberg B. A comparison of treatment modes in the management of myofascial pain dysfunction syndrome. Biofeedback Self Regul. 1986;11(4):279-91. Epub 1986/12/01. PubMed PMID: 3607094.

5. Dahlstrom L. Conservative treatment of mandibular dysfunction. Clinical, experimental and electromyographic studies of biofeedback and occlusal appliances. Swed Dent J Suppl. 1984;24:1-45. Epub 1984/01/01. PubMed PMID: 6395416.

6. de Felicio CM, Melchior MD, da Silva M. Effects of Orofacial Myofunctional Therapy on Temporomandibular Disorders. Cranio-the Journal of Craniomandibular Practice. 2010;28(4):249-59. PubMed PMID: WOS:000293614300006.

7. Dworkin SF, Huggins KH, Wilson L, Mancl L, Turner J, Massoth D, et al. A randomized clinical trial using research diagnostic criteria for temporomandibular disorders-axis II to target clinic cases for a tailored self-care TMD treatment program. J Orofac Pain. 2002;16(1):48-63. Epub 2002/03/14. PubMed PMID: 11889659.

8. Jokstad A, Mo A, Krogstad BS. Clinical comparison between two different splint designs for temporomandibular disorder therapy. Acta Odontologica Scandinavica. 2005;63(4):218-26. PubMed PMID: 41074367.

9. List T, Helkimo M. Acupuncture and occlusal splint therapy in the treatment of craniomandibular disorders. II. A 1-year follow-up study. Acta odontologica Scandinavica. 1992;50(6):375-85. PubMed PMID: 23849805.

10. Mejersjo C, Wenneberg B. Diclofenac sodium and occlusal splint therapy in TMJ osteoarthritis: A randomized controlled trial. Journal of Oral Rehabilitation. 2008;35(10):729-38. PubMed PMID: 352290022.

11. Michelotti A, Iodice G, Vollaro S, Steenks MH, Farella M. Evaluation of the short-term effectiveness of education versus an occlusal splint for the treatment of myofascial pain of the jaw muscles. J Am Dent Assoc. 2012;143(1):47-53. Epub 2011/12/31. PubMed PMID: 22207667.

12. Minakuchi H, Kuboki T, Maekawa K, Matsuka Y, Yatani H. Self-reported remission, difficulty, and satisfaction with nonsurgical therapy used to treat anterior disc displacement without reduction. Oral Surg Oral Med Oral Pathol Oral Radiol Endod. 2004;98(4):435-40. Epub 2004/10/09. doi: 10.1016/s1079210403007029. PubMed PMID: 15472659.

13. Qvintus V, Suominen AL, Huttunen J, Raustia A, Ylostalo P, Sipila K. Efficacy of stabilisation splint treatment on facial pain-1-year follow-up. Journal of Oral Rehabilitation. 2015;42(6):439-46. doi: 10.1111/joor.12275. PubMed PMID: WOS:000353393800006.

14. Raustia AM. Diagnosis and treatment of temporomandibular joint dysfunction. Advantages of computed tomography diagnosis. Stomatognathic treatment and acupuncture--a randomized trial. Proc Finn Dent Soc. 1986;82 Suppl 9-10:1-41. Epub 1986/01/01. PubMed PMID: 3562447.

15. Tavera AT, Montoya MCP, Calderon E, Gorodezky G, Wixtrom RN. Approaching Temporomandibular Disorders From a New Direction: A Randomized Controlled Clinical Trial of the TMDes (TM) Ear System. Cranio-the Journal of Craniomandibular Practice. 2012;30(3):172-82. PubMed PMID: WOS:000306616800004.

**Not adequate control group**

1. Al-Rafah EM, Alammari MR, Banasr FH. The efficacy of bilateral balanced and canine guidance occlusal splints in the treatment of temporomandibular joint disorder. Oral Health Dent Manag. 2014;13(2):536-42. Epub 2014/07/06. PubMed PMID: 24984678.

2. Costa YM, Porporatti AL, Stuginski-Barbosa J, Bonjardim LR, Speciali JG, Conti PCR. Headache attributed to masticatory myofascial pain: impact on facial pain and pressure pain threshold. Journal of Oral Rehabilitation. 2016;43(3):161-8. doi: 10.1111/joor.12357. PubMed PMID: WOS:000370488000001.

3. Davies SJ, Gray RJ. The pattern of splint usage in the management of two common temporomandibular disorders. Part II: The stabilisation splint in the treatment of pain dysfunction syndrome. Br Dent J. 1997;183(7):247-51. Epub 1997/11/19. PubMed PMID: 9364091.

4. Elsharkawy TM, Ali NM. Evaluation of acupuncture and occlusal splint therapy in the treatment of temporomandibular joint disorders. Egypt Dent J. 1995;41(3):1227-32. Epub 1995/07/01. PubMed PMID: 9497660.

5. Ferreira LA, De Oliveira RG, Guimaraes JP, Carvalho ACP, De Paula MVQ. Laser acupuncture in patients with temporomandibular dysfunction: A randomized controlled trial. Lasers in Medical Science. 2013;28(6):1549-58. PubMed PMID: 370326946.

6. Ferreira LA, Grossmann E, Januzzi E, Goncalves R, Mares FAG, de Paula MVQ, et al. Ear Acupuncture Therapy for Masticatory Myofascial and Temporomandibular Pain: A Controlled Clinical Trial. Evidence-Based Complementary and Alternative Medicine. 2015. doi: 10.1155/2015/342507. PubMed PMID: WOS:000360495800001.

7. Forssell H, Kirveskari P, Kangasniemi P. Effect of occlusal adjustment on mandibular dysfunction. A double-blind study. Acta Odontol Scand. 1986;44(2):63-9. Epub 1986/04/01. PubMed PMID: 3524093.

8. Glaros AG, Owais Z, Lausten L. Reduction in parafunctional activity: A potential mechanism for the effectiveness of splint therapy. Journal of Oral Rehabilitation. 2007;34(2):97-104. PubMed PMID: 46146600.

9. Hamata MM, Zuim PR, Garcia AR. Comparative evaluation of the efficacy of occlusal splints fabricated in centric relation or maximum intercuspation in temporomandibular disorders patients. J Appl Oral Sci. 2009;17(1):32-8. Epub 2009/01/17. PubMed PMID: 19148403; PubMed Central PMCID: PMCPMC4327611.

10. Landry ML, Rompre PH, Manzini C, Guitard F, de Grandmont P, Lavigne GJ. Reduction of sleep bruxism using a mandibular advancement device: an experimental controlled study. Int J Prosthodont. 2006;19(6):549-56. Epub 2006/12/15. PubMed PMID: 17165292.

11. Machon V, Hirjak D, Lukas J. Therapy of the osteoarthritis of the temporomandibular joint. J Craniomaxillofac Surg. 2011;39(2):127-30. Epub 2010/08/10. doi: 10.1016/j.jcms.2010.04.010. PubMed PMID: 20692843.

12. Maloney GE, Mehta N, Forgione AG, Zawawi KH, Al-Badawi EA, Driscoll SE. Effect of a passive jaw motion device on pain and range of motion in TMD patients not responding to flat plane intraoral appliances. Cranio. 2002;20(1):55-66. Epub 2002/02/08. PubMed PMID: 11831346.

13. Manns A, Miralles R, Cumsille F. Influence of vertical dimension on masseter muscle electromyographic activity in patients with mandibular dysfunction. J Prosthet Dent. 1985;53(2):243-7. Epub 1985/02/01. PubMed PMID: 3856667.

14. Manns A, Miralles R, Santander H, Valdivia J. Influence of the vertical dimension in the treatment of myofascial pain-dysfunction syndrome. J Prosthet Dent. 1983;50(5):700-9. Epub 1983/11/01. PubMed PMID: 6580439.

15. Ozkan F, Cakir Ozkan N, Erkorkmaz U. Trigger point injection therapy in the management of myofascial temporomandibular pain. Agri. 2011;23(3):119-25. PubMed PMID: 362480190.

16. Pho Duc JM, Huning SV, Grossi ML. Parallel Randomized Controlled Clinical Trial in Patients with Temporomandibular Disorders Treated with a CAD/CAM Versus a Conventional Stabilization Splint. Int J Prosthodont. 2016;29(4):340-50. Epub 2016/08/02. doi: 10.11607/ijp.4711. PubMed PMID: 27479339.

17. Rizzatti-Barbosa CM, Martinelli DA, Ambrosano GMB, de Albergaria-Barbosa JR. Therapeutic response of benzodiazepine, orphenadrine citrate and occlusal splint association in TMD pain. Cranio-the Journal of Craniomandibular Practice. 2003;21(2):116-20. PubMed PMID: WOS:000182166100007.

18. Rohida NS, Bhad W. A clinical, MRI, and EMG analysis comparing the efficacy of twin blocks and flat occlusal splints in the management of disc displacements with reduction. World J Orthod. 2010;11(3):236-44. Epub 2010/09/30. PubMed PMID: 20877732.

19. Schmitter M, Zahran M, Duc JMP, Henschel V, Rammelsberg P. Conservative therapy in patients with anterior disc displacement without reduction using 2 common splints: A randomized clinical trial. Journal of Oral and Maxillofacial Surgery. 2005;63(9):1295-303. doi: 10.1016/j.joms.2005.05.294. PubMed PMID: WOS:000231639200008.

20. Vallon D, Nilner M, Soderfeldt B. Treatment outcome in patients with craniomandibular disorders of muscular origin: a 7-year follow-up. J Orofac Pain. 1998;12(3):210-8. Epub 1998/10/22. PubMed PMID: 9780942.

21. van der Glas HW, Buchner R, van Grootel RJ. [Comparison of treatment options for myogenous temporomandibular dysfunction]. Ned Tijdschr Tandheelkd. 2000;107(12):505-12. Epub 2001/06/01. PubMed PMID: 11383262.

22. Wenneberg B, Nystrom T, Carlsson GE. Occlusal equilibration and other stomatognathic treatment in patients with mandibular dysfunction and headache. J Prosthet Dent. 1988;59(4):478-83. Epub 1988/04/01. PubMed PMID: 3162993.

**No adequate population**

1. Baad-Hansen L, Jadidi F, Castrillon E, Thomsen PB, Svensson P. Effect of a nociceptive trigeminal inhibitory splint on electromyographic activity in jaw closing muscles during sleep. J Oral Rehabil. 2007;34(2):105-11. Epub 2007/01/25. doi: 10.1111/j.1365-2842.2006.01717.x. PubMed PMID: 17244232.

2. Cane L, Schieroni MP, Ribero G, Ferrero M, Carossa S. Effectiveness of the Michigan splint in reducing functional cervical disturbances: a preliminary study. Cranio. 1997;15(2):132-5. Epub 1997/04/01. PubMed PMID: 9586515.

3. Dalewski B, Chrusciel-Nogalska M, Frazczak B. Occlusal splint versus modified nociceptive trigeminal inhibition splint in bruxism therapy: a randomized, controlled trial using surface electromyography. Australian Dental Journal. 2015;60(4):445-54. doi: 10.1111/adj.12259. PubMed PMID: WOS:000367692000006.

4. Evcik D, Kavuncu V, Cakir T, Subasi V, Yaman M. Laser therapy in the treatment of carpal tunnel syndrome: A randomized controlled trial. Photomedicine and Laser Surgery. 2007;25(1):34-9. doi: 10.1089/pho.2006.2032. PubMed PMID: WOS:000244984300007.

5. Fischer MJ, Reiners A, Kohnen R, Bernateck M, Gutenbrunner C, Fink M, et al. Do occlusal splints have an effect on complex regional pain syndrome? A randomized, controlled proof-of-concept trial. Clin J Pain. 2008;24(9):776-83. Epub 2008/10/22. doi: 10.1097/AJP.0b013e3181790355. PubMed PMID: 18936595.

6. Goncalves DAG, Camparis CM, Speciali JG, Castanharo SM, Ujikawa LT, Lipton RB, et al. Treatment of Comorbid Migraine and Temporomandibular Disorders: A Factorial, Double-Blind, Randomized, Placebo-Controlled Study. Journal of Orofacial Pain. 2013;27(4):325-35. PubMed PMID: WOS:000326428200005.

7. Magnusson T, Syren M. Therapeutic jaw exercises and interocclusal appliance therapy. A comparison between two common treatments of temporomandibular disorders. Swed Dent J. 1999;23(1):27-37. Epub 1999/06/17. PubMed PMID: 10371003.

8. Molina-Torres G, Rodriguez-Archilla A, Mataran-Penarrocha G, Albornoz-Cabello M, Aguilar-Ferrandiz ME, Castro-Sanchez AM. Laser Therapy and Occlusal Stabilization Splint for Temporomandibular Disorders in Patients With Fibromyalgia Syndrome: A Randomized, Clinical Trial. Altern Ther Health Med. 2016;22(5):23-31. Epub 2016/09/14. PubMed PMID: 27622957.

9. Raphael KG, Marbach JJ. Widespread pain and the effectiveness of oral splints in myofascial face pain. J Am Dent Assoc. 2001;132(3):305-16. Epub 2001/03/22. PubMed PMID: 11258087.

10. Raphael KG, Marbach JJ, Klausner JJ, Teaford MF, Fischoff DK. Is bruxism severity a predictor of oral splint efficacy in patients with myofascial face pain? Journal of oral rehabilitation. 2003;30(1):17-29. PubMed PMID: 35479879.

11. Roark AL, Glaros AG, O'Mahony AM. Effects of interocclusal appliances on EMG activity during parafunctional tooth contact. J Oral Rehabil. 2003;30(6):573-7. Epub 2003/06/06. PubMed PMID: 12787453.

12. Shankland WE. Nociceptive trigeminal inhibition--tension suppression system: a method of preventing migraine and tension headaches. Compend Contin Educ Dent. 2002;23(2):105-8, 10, 12-3; quiz 14. Epub 2002/03/28. PubMed PMID: 11915652.

13. Wahlund K. Temporomandibular disorders in adolescents. Epidemiological and methodological studies and a randomized controlled trial. Swedish dental journal. 2003;Supplement.(164):inside front cover, 2-64. PubMed PMID: 38243974.

14. Wahlund K, List T, Larsson B. Treatment of temporomandibular disorders among adolescents: A comparison between occlusal appliance, relaxation training, and brief information. Acta Odontologica Scandinavica. 2003;61(4):203-11. PubMed PMID: 37070843.

15. Wahlund K, Nilsson IM, Larsson B. Treating temporomandibular disorders in adolescents: a randomized, controlled, sequential comparison of relaxation training and occlusal appliance therapy. Journal of oral & facial pain and headache. 2015;29(1):41-50. PubMed PMID: 603210354.

**Not adequate splint therapy**

1. Berguer A, Kovacs F, Abraira V, Mufraggi N, Royuela A, Muriel A, et al. Neuro-reflexotherapy for the management of myofascial temporomandibular joint pain: a double-blind, placebo-controlled, randomized clinical trial. J Oral Maxillofac Surg. 2008;66(8):1664-77. Epub 2008/07/19. doi: 10.1016/j.joms.2008.01.049. PubMed PMID: 18634956.

2. Conti PCR, Correa ASD, Lauris JRP, Stuginski-Barbosa J. Management of painful temporomandibular joint clicking with different intraoral devices and counseling: a controlled study. Journal of Applied Oral Science. 2015;23(5):529-35. doi: 10.1590/1678-775720140438. PubMed PMID: WOS:000364646200013.

3. Davies SJ, Gray RJ. The pattern of splint usage in the management of two common temporomandibular disorders. Part I: The anterior repositioning splint in the treatment of disc displacement with reduction. Br Dent J. 1997;183(6):199-203. Epub 1997/11/05. PubMed PMID: 9345797.

4. DeVocht JW, Goertz CM, Hondras MA, Long CR, Schaeffer W, Thomann L, et al. A pilot study of a chiropractic intervention for management of chronic myofascial temporomandibular disorder. J Am Dent Assoc. 2013;144(10):1154-63. Epub 2013/10/02. PubMed PMID: 24080932; PubMed Central PMCID: PMCPMC4103021.

5. Madani AS, Mirmortazavi A. Comparison of three treatment options for painful temporomandibular joint clicking. J Oral Sci. 2011;53(3):349-54. Epub 2011/10/01. PubMed PMID: 21959663.

6. Nagata K, Maruyama H, Mizuhashi R, Morita S, Hori S, Yokoe T, et al. Efficacy of stabilisation splint therapy combined with non-splint multimodal therapy for treating RDC/TMD axis I patients: a randomised controlled trial. J Oral Rehabil. 2015;42(12):890-9. Epub 2015/07/16. doi: 10.1111/joor.12332. PubMed PMID: 26174571.

7. Naikmasur V, Bhargava P, Guttal K, Burde K. Soft occlusal splint therapy in the management of myofascial pain dysfunction syndrome: A follow-up study. Indian Journal of Dental Research. 2008;19(3):196-203. PubMed PMID: 352413939.

8. Nilsson H, Ekberg E. Do psychological factors and general health influence the short-term efficacy of resilient appliance therapy in patients with temporomandibular disorder pain? Acta Odontol Scand. 2010;68(3):141-7. Epub 2010/02/02. doi: 10.3109/00016350903514418. PubMed PMID: 20113145.

9. Nilsson H, Limchaichana N, Nilner M, Ekberg EC. Short-term treatment of a resilient appliance in TMD pain patients: A randomized controlled trial. Journal of Oral Rehabilitation. 2009;36(8):547-55. PubMed PMID: 354957128.

10. Nilsson H, Vallon D, Ekberg EC. Long-term efficacy of resilient appliance therapy in TMD pain patients: a randomised, controlled trial. J Oral Rehabil. 2011;38(10):713-21. Epub 2011/03/26. doi: 10.1111/j.1365-2842.2011.02210.x. PubMed PMID: 21434963.

11. Rampello A, Saccucci M, Falisi G, Panti F, Polimeni A, Di Paolo C. A new aid in temporomandibular joint disorders' therapy: The universal neuromuscular immediate relaxing appliance. Journal of Biological Regulators and Homeostatic Agents. 2013;27(4):1011-9. PubMed PMID: 372269931.

12. Schiffman EL, Look JO, Hodges JS, Swift JQ, Decker KL, Hathaway KM, et al. Randomized effectiveness study of four therapeutic strategies for TMJ closed lock. J Dent Res. 2007;86(1):58-63. Epub 2006/12/26. PubMed PMID: 17189464; PubMed Central PMCID: PMCPMC2278036.

13. Tecco S, Caputi S, Tete S, Orsini G, Festa F. Intra-articular and muscle symptoms and subjective relief during TMJ internal derangement treatment with maxillary anterior repositioning splint or SVED and MORA splints: A comparison with untreated control subjects. Cranio. 2006;24(2):119-29. Epub 2006/05/23. doi: 10.1179/crn.2006.019. PubMed PMID: 16711274.

14. Vicente-Barrero M, Yu-Lu SL, Zhang BX, Bocanegra-Perez S, Duran-Moreno D, Lopez-Marquez A, et al. The efficacy of acupuncture and decompression splints in the treatment of temporomandibular joint pain-dysfunction syndrome. Medicina Oral Patologia Oral Y Cirugia Bucal. 2012;17(6):E1028-E33. doi: 10.4317/medoral.17567. PubMed PMID: WOS:000314401800019.

15. Weggen T, Schindler HJ, Kordass B, Hugger A. Clinical and electromyographic follow-up of myofascial pain patients treated with two types of oral splint: a randomized controlled pilot study. Int J Comput Dent. 2013;16(3):209-24. Epub 2013/12/25. PubMed PMID: 24364193.

16. Williamson EH, Rosenzweig BJ. The treatment of temporomandibular disorders through repositioning splint therapy: a follow-up study. Cranio : the journal of craniomandibular practice. 1998;16(4):222-5. PubMed PMID: 129395820.

**Not RCTs**

1. Aksakalli S, Temucin F, Pamukcu A, Ezirganli S, Kazancioglu HO, Malkoc MA. Effectiveness of two different splints to treat temporomandibular disorders. J Orofac Orthop. 2015;76(4):318-27. Epub 2015/07/08. doi: 10.1007/s00056-015-0294-4. PubMed PMID: 26149971.

2. Al-Saad M, Akeel R. EMG and pain severity evaluation in patients with TMD using two different occlusal devices. International Journal of Prosthodontics. 2001;14(1):15-21. PubMed PMID: WOS:000166886300003.

3. Brown DT, Gaudet Jr EL. Outcome measurement for treated and untreated TMD patients using the TMJ scale. Cranio : the journal of craniomandibular practice. 1994;12(4):216-22. PubMed PMID: 125018166.

4. Cawley R. A comparison of two splints in the treatment of TMJ pain dysfunction syndrome. Br Dent J. 1991;170(7):254. Epub 1991/04/06. PubMed PMID: 2025457.

5. Daif E. Correlation of splint therapy outcome with the electromyography of masticatory muscles in temporomandibular disorder with myofascial pain. Pain Practice. 2016;16:87. PubMed PMID: 72302594.

6. Davies SJ, Gray RJ. The pattern of splint usage in the management of two common temporomandibular disorders. Part III: Long-term follow-up in an assessment of splint therapy in the management of disc displacement with reduction and pain dysfunction syndrome. British dental journal. 1997;183(8):279-83. PubMed PMID: 127331955.

7. de Felicio CM, Mazzetto MO, de Silva MA, Bataglion C, Hotta TH. A preliminary protocol for multi-professional centers for the determination of signs and symptoms of temporomandibular disorders. Cranio. 2006;24(4):258-64. Epub 2006/11/08. doi: 10.1179/crn.2006.041. PubMed PMID: 17086855.

8. de Leeuw JR, Ros WJ, Steenks MH, Lobbezoo-Scholte AM, Bosman F, Winnubst JA. Craniomandibular dysfunction: patient characteristics related to treatment outcome. J Oral Rehabil. 1994;21(6):667-78. Epub 1994/11/01. PubMed PMID: 7830202.

9. de Leeuw JR, Steenks MH, Ros WJ, Lobbezoo-Scholte AM, Bosman F, Winnubst JA. Assessment of treatment outcome in patients with craniomandibular dysfunction. J Oral Rehabil. 1994;21(6):655-66. Epub 1994/11/01. PubMed PMID: 7830201.

10. Demirkol N, Sari F, Bulbul M, Demirkol M, Simsek I, Usumez A. Effectiveness of occlusal splints and low-level laser therapy on myofascial pain. Lasers in Medical Science. 2014:1-6. PubMed PMID: 52994443.

11. Garefis P, Grigoriadou E, Zarifi A, Koidis PT. Effectiveness of conservative treatment for craniomandibular disorders: a 2-year longitudinal study. J Orofac Pain. 1994;8(3):309-14. Epub 1994/01/01. PubMed PMID: 7812229.

12. Gavish A, Winocur E, Ventura YS, Halachmi M, Gazit E. Effect of stabilization splint therapy on pain during chewing in patients suffering from myofascial pain. Journal of oral rehabilitation. 2002;29(12):1181-6. PubMed PMID: 35546865.

13. Glaros AG. Temporomandibular disorders and facial pain: a psychophysiological perspective. Appl Psychophysiol Biofeedback. 2008;33(3):161-71. Epub 2008/08/30. doi: 10.1007/s10484-008-9059-9. PubMed PMID: 18726689.

14. Gray RJ, Quayle AA, Davies SJ. 'A comparison of two splints in the treatment of TMJ pain dysfunction syndrome'. Br Dent J. 1991;170(10):366. Epub 1991/05/25. PubMed PMID: 2064856.

15. Inchingolo F, Tatullo M, Marrelli M, Inchingolo AM, Tarullo A, Inchingolo AD, et al. Combined occlusal and pharmacological therapy in the treatment of temporo-mandibular disorders. Eur Rev Med Pharmacol Sci. 2011;15(11):1296-300. Epub 2011/12/27. PubMed PMID: 22195362.

16. Konstantinovic VS, Lazic V. Occlusion splint therapy in patients with craniomandibular disorders (CMD). Journal of Craniofacial Surgery. 2006;17(3):572-8. PubMed PMID: 44314861.

17. Mazzeto MO, Hotta TH, Mazzetto RG. Analysis of TMJ vibration sounds before and after use of two types of occlusal splints. Braz Dent J. 2009;20(4):325-30. Epub 2010/01/14. PubMed PMID: 20069257.

18. McCreary CP, Clark GT, Oakley ME, Flack V. Predicting response to treatment for temporomandibular disorders. J Craniomandib Disord. 1992;6(3):161-9. Epub 1992/01/01. PubMed PMID: 1401133.

19. Mortazavi SH, Motamedi MH, Navi F, Pourshahab M, Bayanzadeh SM, Hajmiragha H, et al. Natl J Maxillofac Surg. 2010;1(2):108-11. Epub 2010/07/01. doi: 10.4103/0975-5950.79210. PubMed PMID: 22442579; PubMed Central PMCID: PMCPMC3304195.

20. Nemcovsky CE, Gazit E, Serfati V, Gross M. A comparative study of three therapeutic modalities in a temporomandibular disorder (TMD) population. Cranio. 1992;10(2):148-55; discussion 56-7. Epub 1992/04/01. PubMed PMID: 1423673.

21. Ohnuki T, Fukuda M, Nakata A, Nagai H, Takahashi T, Sasano T, et al. Evaluation of the position, mobility, and morphology of the disc by MRI before and after four different treatments for temporomandibular joint disorders. Dentomaxillofac Radiol. 2006;35(2):103-9. Epub 2006/03/22. doi: 10.1259/dmfr/25020275. PubMed PMID: 16549437.

22. Patel K, Hemmings KW, Vaughan S. The provision of occlusal splints in primary dental care. Prim Dent Care. 2000;7(3):109-13. Epub 2001/06/19. PubMed PMID: 11404899.

23. Pettengill CA, Growney MR, Jr., Schoff R, Kenworthy CR. A pilot study comparing the efficacy of hard and soft stabilizing appliances in treating patients with temporomandibular disorders. J Prosthet Dent. 1998;79(2):165-8. Epub 1998/03/26. PubMed PMID: 9513102.

24. Sato S, Kawamura H, Motegi K. Management of nonreducing temporomandibular joint disk displacement. Evaluation of three treatments. Oral Surg Oral Med Oral Pathol Oral Radiol Endod. 1995;80(4):384-8. Epub 1995/10/01. PubMed PMID: 8521099.

25. Scopel V, Alves Da Costa GS, Urias D. An electromyographic study of masseter and anterior temporalis muscles in extra-articular myogenous TMJ pain patients compared to an asymptomatic and normal population. Cranio. 2005;23(3):194-203. PubMed PMID: 41175873.

26. Seifeldin SA, Elhayes KA. Soft versus hard occlusal splint therapy in the management of temporomandibular disorders (TMDs). Saudi Dental Journal. 2015;27(4):208-14. PubMed PMID: 605168755.

27. Stiesch-Scholz M, Fink M, Tschernitschek H, Rossbach A. Medical and physical therapy of temporomandibular joint disk displacement without reduction. Cranio. 2002;20(2):85-90. Epub 2002/05/11. PubMed PMID: 12002834.

28.Winocur E, Gavish A, Emodi-Perlman A, Halachmi M, Eli I. Hypnorelaxation as treatment for myofascial pain disorder: a comparative study. Oral surgery, oral medicine, oral pathology, oral radiology, and endodontics. 2002;93(4):429-34. PubMed PMID: 35601935.

29. Williamson EH, Navarro EZ, Zwemer JD. A comparison of electromyographic activity between anterior repositioning splint therapy and a centric relation splint. Cranio : the journal of craniomandibular practice. 1993;11(3):178-83. PubMed PMID: 23944810.

**Other reasons**

1. Bergstrom I, List T, Magnusson T. A follow-up study of subjective symptoms of temporomandibular disorders in patients who received acupuncture and/or interocclusal appliance therapy 18-20 years earlier. Acta Odontol Scand. 2008;66(2):88-92. Epub 2008/05/01. doi: 10.1080/00016350801978660. PubMed PMID: 18446549.

2. Erixon CL, Exberg E. Self-perceived effects of occlusal appliance therapy on TMD patients: An eight-year follow-up. Swedish Dental Journal. 2013;37(1):13-23. PubMed PMID: 368953670.

3. Giannakopoulos NN, Katsikogianni EN, Hellmann D, Eberhard L, Leckel M, Schindler HJ, et al. Comparison of three different options for immediate treatment of painful temporomandibular disorders: a randomized, controlled pilot trial. Acta Odontol Scand. 2016;74(6):480-6. Epub 2016/07/14. doi: 10.1080/00016357.2016.1204558. PubMed PMID: 27410169.

4. List T, Helkimo M, Andersson S, Carlsson GE. Acupuncture and occlusal splint therapy in the treatment of craniomandibular disorders. Part I. A comparative study. Swedish dental journal. 1992;16(4):125-41. PubMed PMID: 23847119.
