# Supplementary material for: Occlusal stabilization splint for patients with temporomandibular disorders: Meta-analysis of short and long term effects
Source: PLoS One. 2017 Feb 6;12(2):e0171296. doi: 10.1371/journal.pone.0171296 (PMC5293221; doi:10.1371/journal.pone.0171296)
Supplement: S1 Table — (DOC) [file pone.0171296.s012.doc]

**S1 Table.** Descriptive characteristics of included studies

| Study characteristic | | | Population characteristics | | Intervention characteristics | | | | | Outcome characteristics | Quality assess-ment |
| --- | --- | --- | --- | --- | --- | --- | --- | --- | --- | --- | --- |
| Studies | Study design | Follow up  period | Patients  total n (m/f) | Age | TMD diagnostic criteria | Origin of TMD | Control | Study group | Control | Outcomes | Jadad score |
| Raustia  1985 [47] | RCT | 3 mos. | 50  (11M/39F) | 27.8/ 26.4 | Clinical exam | muscular and articular | physical therapy | 25 | 25 | pain reduction | 2 |
| Dahlstrom 1985 [41] | RCT | 3 mos. | 19 (19F) | 26.3  range 17-71 | Clinical exam | muscular | occlusal appliances | 10 | 9 | pain reduction | 2 |
| Rubinoff  1987 [33] | RCT | NA | 28 (4M/24F) | 33.7  range 18-62 | Clinical exam | muscular | non-occluding splint | 15 | 13 | pain reduction maximum mouth opening | 4 |
| Johansson  1991 [50] | RCT | 3 mos. | 45 | NA | Clinical exam | muscular | I -physical therapy II-no treatment | 15 | I -15 II-15 | pain reduction | 2 |
| Gray  1991 [42] | RCT | 3 mos. | 55 (7M/48F) | 28.1  range 16-50 | Clinical exam | muscular | occlusal appliances | 34 | 21 | pain reduction | 3 |
| Lundh 1992 [57] | RCT | 12 mos. | 51 (5M/46F) | 29  range 14-61 | Clinical exam and arthrographic | articular | no treatment | 25 | 26 | pain reduction | 3 |
| List  1993 [51] | RCT | 3 mos. | 47 | range 22-69 | Clinical exam | muscular and articular | I - no treatment II-physical therapy | 18 | I- 9 II - 20 | pain intensity (VAS) | 2 |
| Turk 1993[54] | RCT | 6 wks.  6 mos. | 78 (14M/66F) | 34.1 (18-55) | Clinical exam | muscular | I – behavioral  II- no treatment | 28 | I-30  II- 20 | pain intensity (PSS) depression (CES-D) | 2 |
| Dao  1994 [34] | RCT | 10 wks. | 41  (8M/33F) | 29.3/27.9 range 16-40 | RDC/TMD | muscular | non-occluding splint | 22 | 19 | pain reduction | 2 |
| Ekberg 1998 [35] | RCT | 10 wks. | 60 (5M/55F) | 30 (13-76) | Clinical exam | articular | non-occluding splint | 30 | 30 | pain reduction  muscle tenderness reduction TMJ tenderness reduction | 5 |

RCT- Randomized controlled trial; NA- not available; RDC/TMD- Research diagnostic criteria of TMD; VAS- Visual Analogue Scale; PSS-Pain Severity Scale; CPI-Characteristic pain intensity; NRS-Numeric rating scale, CES-D- Center for Epidemiologic Studies-Depression scale; SCL-90R -Modified Symptom Checklist 90-revised

**S1Table.** Continued

| Study characteristic | | | Population characteristics | | Intervention characteristics | | | | | Outcome characteristics | Quality assess-ment |
| --- | --- | --- | --- | --- | --- | --- | --- | --- | --- | --- | --- |
| Studies | Study design | Follow up  period | Patients  total n (m/f) | Age | TMD diagnostic criteria | Origin of TMD | Control | Study group | Control | Outcomes | Jadad score |
| Carlson 2001 [52] | RCT | 6 wks.  26 wks. | 44 (10M/34F) | 34.6 | RDC/TMD | muscular | behavioral therapy | 23 | 21 | pain intensity (VAS,PSS)  depression (SCL-90R) | 3 |
| Ekberg 2002 [36] | RCT | 12 mos. | 35 | 30 (13-76) | Clinical exam | articular | non-occluding splint | 27 | 8 | 50% reduction of pain | 5 |
| Kuttila 2002 [38] | RCT | 10 wks. | 34 (9M/25F) | 45/47  range 25-65 | Criteria by De Leeuw | muscular and articular | non-occluding splint | 18 | 16 | pain reduction muscle tenderness reduction TMJ tenderness reduction | 5 |
| Ekberg  2003 [37] | RCT | 10 wks. | 60 (8M/52F) | 31/28 | RDC/TMD | muscular | non-occluding splint | 30 | 30 | pain reduction  pain intensity (VAS)  muscle tenderness reduction TMJ tenderness reduction | 3 |
| Ekberg  2004 [19] | RCT | 12 mos. | 40 | NA | RDC/TMD | muscular | non-occluding splint | 30 | 10 | pain reduction | 3 |
| Wassell  2004 [39] | RCT | 6 wks. | 72 (9M/63F) | 37.9/35.9 | International Headache Society | muscular and articular | non-occluding splint | 34 | 38 | pain intensity (VAS) maximum mouth opening | 3 |
| Stiesch-Scholz 2005[43] | RCT | 3 mos. | 40 (5M/35F) | 34.8/32.5 range 18-62 | Clinical exam and MRI | articular | occlusal appliances | 20 | 20 | pain intensity (VAS), muscle tenderness reduction TMJ tenderness reduction, maximum mouth opening | 3 |

RCT- Randomized controlled trial; NA- not available; RDC/TMD- Research diagnostic criteria of TMD; VAS- Visual Analogue Scale; PSS-Pain Severity Scale; CPI-Characteristic pain intensity; NRS-Numeric rating scale, CES-D- Center for Epidemiologic Studies-Depression scale; SCL-90R -Modified Symptom Checklist 90-revised

**S1 Table.** Continued

| Study characteristic | | | Population characteristics | | Intervention characteristics | | | | | Outcome characteristics | Quality assess-ment |
| --- | --- | --- | --- | --- | --- | --- | --- | --- | --- | --- | --- |
| Studies | Study design | Follow up  period | Patients  total n (m/f) | Age | TMD diagnostic criteria | Origin of TMD | Control | Study group | Control | Outcomes | Jadad score |
| Al Quran  2006 [44] | RCT | 3 mos. | 114 (49M/65F) | 33.5  range 15-62 | Clinical exam | muscular | I - occlusal appliances II-no treatment | 38 | I -38 II-38 | pain reduction pain intensity (VAS) | 2 |
| Truelove 2006[46] | RCT | 3 mos.  12 mos. | 200 (28M/  172F) | 36/35/36 | RDC/TMD | muscular and articular | I - occlusal appliances II-minimal treatment | 68 | I-68  II- 64 | pain intensity (CPI) | 3 |
| Wassell  2006 [40] | RCT | 12 mos. | 39 | 19-65 | International Headache Society | muscular and articular | non-occluding splint | 27 | 12 | pain intensity (VAS) maximum mouth opening | 3 |
| Glaros 2007[55] | RCT | 1 mo.  12 mos. | 8 F | 39/32.8 | RDC/TMD | muscular and articular | behavioral therapy | 4 | 4 | pain intensity (NRS) | 2 |
| Ismail 2007 [49] | RCT | 12 wks. | 26 (3M/23F) | 44.5/41.7 | RDC/TMD and MRI | articular | physical therapy | 13 | 13 | pain intensity (VAS)  maximum mouth opening | 3 |
| Nilner 2008 [18] | RCT | 10 wks. | 65 (7M/58F) | 36/37  range 18-71 | RDC/TMD | muscular and articular | occlusal appliances | 33 | 32 | 50% reduction of pain | 3 |
| Oz  2010 [48] | RCT | 3 mos. | 40 (6M/34F) | 32.84 | RDC/TMD | muscular | physical therapy | 20 | 20 | pain reduction maximum mouth opening depression (SCL-90R) | 5 |
| Conti  2012 [45] | RCT | 3 mos. | 39 (5M/34F) | 38.1/35.2/  38.1 | RDC/TMD | muscular | I - occlusal appliances II-counseling | 17 | I -13 II- 9 | pain reduction | 2 |

RCT- Randomized controlled trial; NA- not available; RDC/TMD- Research diagnostic criteria of TMD; VAS- Visual Analogue Scale; PSS-Pain Severity Scale; CPI-Characteristic pain intensity; NRS-Numeric rating scale, CES-D- Center for Epidemiologic Studies-Depression scale; SCL-90R -Modified Symptom Checklist 90-revised

**S1 Table.** Continued

| Study characteristic | | | Population characteristics | | Intervention characteristics | | | | | Outcome characteristics | Quality assess-ment |
| --- | --- | --- | --- | --- | --- | --- | --- | --- | --- | --- | --- |
| Studies | Study design | Follow up  period | Patients  total n (m/f) | Age | TMD diagnostic criteria | Origin of TMD | Control | Study group | Control | Outcomes | Jadad score |
| Doepel 2012 [20] | RCT | 6 mos. | 52 | ≥ 18 | RDC/TMD | muscular and articular | occlusal appliances | 24 | 28 | 50% reduction of pain depression (SCL-90R) | 3 |
| Daif  2012 [58] | RCT | 6 mos. | 40 (17M/23F) | 32  range 22-46 | Clinical exam | muscular | no treatment | 20 | 20 | pain reduction | 3 |
| Niemela 2012 [56] | RCT | 1 mo. | 76 | 43.2/44.1 | RDC/TMD | muscular and articular | exercise and counseling | 39 | 37 | pain intensity (VAS)  TMJ tenderness reduction maximum mouth opening | 3 |
| Zhang 2013 [17] | RCT | 1 mo. | 36 (12M/24F) | 37.8  range 16-57 | RDC/TMD | muscular | non-occluding splint | 18 | 18 | pain reduction pain intensity (VAS) | 5 |
| Shedden Mora  2013 [53] | RCT | 8 wks.  6 mos. | 56 (13M/43F) | 34.3/36.3 | RDC/TMD | muscular and articular | behavioral therapy | 27 | 29 | pain reduction  pain intensity(CPI)  depression (CES-D) | 3 |
| Christidis 2014 [16] | RCT | 10 wks. 12 mos. | 44 | 19-73 | RDC/TMD | articular | occlusal appliances | 21 | 23 | pain reduction maximum mouth opening depression (SCL-90R) | 3 |
| Katyayan 2014 [21] | RCT | 6 mos. | 80 (18M/62F) | 34.4  range 20-56 | RDC/TMD | muscular and articular | exercise and counseling | 40 | 40 | pain intensity (VAS)  maximum mouth opening | 3 |
| Nitecka Buchta 2014 [59] | RCT | 1 mo. | 65 (19M/46F) | 47 (44-70) | RDC/TMD | muscular | no treatment | 35 | 30 | pain intensity (VAS) | 3 |

RCT- Randomized controlled trial; NA- not available; RDC/TMD- Research diagnostic criteria of TMD; VAS- Visual Analogue Scale; PSS-Pain Severity Scale; CPI-Characteristic pain intensity; NRS-Numeric rating scale, CES-D- Center for Epidemiologic Studies-Depression scale; SCL-90R -Modified Symptom Checklist 90-revised
