# Supplementary material for: Occlusal stabilization splint for patients with temporomandibular disorders: Meta-analysis of short and long term effects
Source: PLoS One. 2017 Feb 6;12(2):e0171296. doi: 10.1371/journal.pone.0171296 (PMC5293221; doi:10.1371/journal.pone.0171296)
Supplement: S2 Table — (PDF) [file pone.0171296.s013.pdf]

S2 Table. GRADE rating system

GRADE rating system evidence profile: Occlusal stabilization splint for patients with temporomandibular disorders: meta-analysis of short and long term effects<br>Bibliography:											
**Quality assessment**							**Summary of findings**				
**№ of participants (studies) Follow-up**	**Risk of bias**	**Inconsistency**	**Indirectness**	**Imprecision**	**Publication bias**	**Overall quality of evidence**	**Study event rates (%)**		**Relative effect (95% CI)**	**Anticipated absolute effects**	
							**With Control group**	**With Stabilization splint**		**Risk with Control group**	**Risk difference with Stabilization splint**
**Pain reduction (follow up: 3 months)**											
848 (16 RCTs)	serious [a]	serious [b]	not serious	not serious	none	⨁⨁◯◯ LOW	199/405 (49.1%)	280/443 (63.2%)	**OR 2.08** (1.19 to 3.63)	49 per 100	**18 more per 100** (4 more to 29 more)
**Pain intensity (follow up: 3 months)**											
1118 (14 RCTs)	serious [c]	serious [d]	not serious	not serious	none	⨁⨁◯◯ LOW	552	566	-	-	SMD **0.33 SD lower** (0.61 lower to 0.05 lower)
**Pain reduction (follow up: range 3 months to 12 months)**											
253 (6 RCTs)	serious [e]	serious [f]	not serious	not serious	none	⨁⨁◯◯ LOW	63/109 (57.8%)	82/144 (56.9%)	**OR 1.04** (0.27 to 3.96)	58 per 100	**1 more per 100** (31 fewer to 27 more)
**Pain intensity (follow up: range 3 months to 12 months)**											
553 (7 RCTs)	serious [g]	serious [h]	not serious	not serious	none	⨁⨁◯◯ LOW	270	283	-	-	SMD **0.03 SD lower** (0.34 lower to 0.29 higher)
**Muscle tenderness reduction (follow up: 3 months)**											
194 (4 RCTs)	not serious	not serious	not serious	serious [i]	none	⨁⨁⨁◯ MODERATE	36/96 (37.5%)	51/98 (52.0%)	**OR 1.97** (1.05 to 3.68)	38 per 100	**17 more per 100** (1 more to 31 more)

**TMJ lateral and posterior tenderness reduction (follow up: 3 months)**											
270<br>(5 RCTs)	serious [j]	not serious	not serious	not serious	none	⊕⊕⊕◯<br>MODERATE	97/133<br>(72.9%)	101/137<br>(73.7%)	**OR 1.05**<br>(0.53 to 2.08)	73 per 100	**1 more per 100**<br>(14 fewer to 12 more)
**Maximum mouth opening (follow up: 3 months)**											
298<br>(7 RCTs)	serious [k]	not serious	not serious	not serious	none	⊕⊕⊕◯<br>MODERATE	139	159	-	-	SMD **0.3 SD lower**<br>(0.59 lower to 0.01 lower)
**Depression (follow up: 3 months)**											
290<br>(5 RCTs)	serious [l]	not serious	not serious	not serious	none	⊕⊕⊕◯<br>MODERATE	145	145	-	-	SMD **0.09 lower**<br>(0.44 lower to 0.27 higher)

CI: Confidence interval; **OR:** Odds ratio; **SMD:** Standardised mean difference

a. Investigators in 11 studies report that did not have blinding of participants; in 7 studies it was unclear allocation concealment and random sequence generation.
b. Presence of heterogeneity p< .0001; I square =66%.
c. Investigators in 12 studies report that did not have blinding of participants; in 3 studies it was unclear allocation concealment and random sequence generation.
d. Presence of heterogeneity p< .00001; I square =80%.
e. Investigators in 5 studies report that did not have blinding of participants; in one study it was unclear allocation concealment and random sequence generation; one study did not have blinding of outcome assessment.
f. Presence of heterogeneity p= .0002; I square =79%.
g. Investigators in 5 studies report that did not have blinding of participants
h. Presence of heterogeneity p= .004; I square =66%.
i. lack of sample size.
j. Investigators in 3 studies report that did not have blinding of participants.
k. Investigators in 5 studies report that did not have blinding of participants; in one study it was unclear allocation concealment and random sequence generation.
l. Investigators in 4 studies report that did not have blinding of participants; in one study it was unclear allocation concealment and random sequence generation; one study did not have blinding of outcome assessment.

References
