# Supplementary material for: Occlusal stabilization splint for patients with temporomandibular disorders: Meta-analysis of short and long term effects
Source: PLoS One. 2017 Feb 6;12(2):e0171296. doi: 10.1371/journal.pone.0171296 (PMC5293221; doi:10.1371/journal.pone.0171296)
Supplement: S3 Table — (DOC) [file pone.0171296.s014.doc]

**S3 Table.** Univariate meta-regression model

| Study | Dependent variable | Number of participants | Rate of women | Age, yrs (average) | Therapy duration (months) | TMDs symptoms duration (months) | Pain intensity on VAS before therapy (average) | Length of wearing the splint  1-night only;  2- 24h | Presence of dropouts  + yes; - no |
| --- | --- | --- | --- | --- | --- | --- | --- | --- | --- |
| Raustia 1985 [47] | OR | 50 | 0.78 | 27.8 | 3 | na | na | na | - |
| Dahlstrom 1985 [41] | OR | 19 | 1.00 | 26.3 | 3 | na | na | 1 | + |
| Rubinoff 1987 [33] | OR | 28 | 0.86 | 33.7 | na | na | na | na | + |
| Johansson 1991 [50] | OR | 45 | na | na | 3 | na | na | na | na |
| Johansson 1991¶ [51] | OR | 45 | na | na | 3 | na | na | na | na |
| Gray 1991 [42] | OR | 55 | 0.87 | 28.1 | 3 | na | na | na | - |
| Dao 1994 [34] | OR | 41 | 0.80 | na | 2.5 | 3 | na | 2 | + |
| Ekberg 1998[35] | OR | 60 | 0.92 | 30 | 2.5 | 24 | 78 | 1 | + |
| Kuttila 2002 [38] | OR | 34 | 0.74 | 34 | 2.5 | na | na | 1 | + |
| Ekberg 2003 [37] | OR; SMD | 60 | 0.87 | na | 2.5 | 36 | 75 | 1 | - |
| Al Quran 2006 [44] | OR; SMD | 114 | 0.57 | 33.5 | 3 | na | 63 | 1 | - |
| Al Quran 2006 ¶[44] | OR; SMD | 114 | 0.57 | 33.5 | 3 | na | 63 | 1 | - |
| Nilner 2008 [18] | OR | 65 | 0.89 | 36 | 2.5 | 3 | na | 1 | + |
| Oz 2010 [48] | OR | 40 | 0.85 | 31.8 | 3 | 8.2 | na | 2 | + |
| Conti 2012 [45] | OR | 39 | 0.83 | 38.1 | 3 | na | 50 | 1 | + |
| Conti 2012 ¶ [45] | OR | 39 | 0.77 | 38.1 | 3 | na | 50 | 1 | + |
| Zhang 2013 [17] | OR; SMD | 36 | 0.67 | 37.8 | 1 | 8.3 | 49 | 2 | - |
| Shedden Mora 2013 [53] | OR;SMD | 56 | 0.77 | 34.3 | 2 | 41 | na | 1 | + |
| Christidis 2014 [16] | OR | 44 | na | 41 | 2.5 | na | na | 1 | + |
| List 1993 [51] | SMD | 47 | 0.98 | 43 | 3 | 6 | na | 1 | + |
| List 1993 ¶ [51] | SMD | 47 | 0.98 | 43 | 3 | 6 | na | 1 | + |
| Carlson 2001 [52] | SMD | 44 | 0.77 | 34.6 | 1.5 | 52.3 | 44.1 | 1 | - |
| Wassell 2004 [39] | SMD | 72 | 0.88 | 37.9 | 2.5 | na | na | 1 | - |
| Stiesch-Scholz 2005 [45] | SMD | 40 | 0.88 | 34.8 | 3 | 4 | 54 | 2 | - |
| Ismail 2007 [49] | SMD | 26 | 0.88 | 44.5 | 3 | na | 42 | 2 | - |
| Niemela 2012 [56] | SMD | 76 | 0.82 | 43.2 | 1 | na | 53 | na | + |
| Nitecka Buchta 2014 [59] | SMD | 65 | 0.71 | 47 | 1 | na | na | na | - |

**S3 Table.** Continued

| Study | Dependent variable | Number of participants | Rate of women | Age, yrs (average) | Therapy duration (months) | TMDs symptoms duration (months) | Pain intensity on VAS before therapy (average) | Length of wearing the splint  1-night only;  2- 24h | Presence of dropouts  + yes; - no |
| --- | --- | --- | --- | --- | --- | --- | --- | --- | --- |
| Glaros 2007 [55] | SMD | 8 | 1.00 | 35.9 | 1 | na | na | 2 | + |
| Truelove 2007 [46] | SMD | 136 | 0.89 | 35.6 | 3 | 63.6 | na | 1 | + |
| Truelove 2007¶ [46] | SMD | 132 | 0.85 | 35.6 | 3 | 63.6 | na | 1 | + |
| Turk 1993 [54] | SMD | 58 | 0.83 | 34.1 | 1.5 | 58.8 | na | 2 | + |
| Turk 1993¶ [54] | SMD | 48 | 0.77 | 34.1 | 1.5 | 58.8 | na | 2 | + |

| Dependent variable | | Rate of women | Age, yrs | Therapy duration | TMDs symptoms duration | Pain intensity on VAS before therapy (average) | Length of wearing the splint | Presence of dropouts |
| --- | --- | --- | --- | --- | --- | --- | --- | --- |
|  |  |  | (average) | (months) | (months) |  | 1-night; | + yes; - no |
|  |  |  |  |  |  |  | 2- 24h |  |
| logarithm OR | coefficient | -1.73 | 0.08 | -0.86 | -0.02 | -0.03 | 1.73 | -0.32 |
|  | SE | 2.65 | 0.08 | 0.63 | 0.03 | 0.04 | 0.69 | 0.61 |
|  | p | 0.515 | 0.325 | 0.172 | 0.503 | 0.434 | 0.011* | 0.598 |
| SMD | coefficient | 2.33 | -0.01 | 0.27 | - | -0.02 | -0.26 | -0.19 |
|  | SE | 1.20 | 0.03 | 0.18 | - | 0.03 | 0.33 | 0.31 |
|  | p | 0.053 | 0.650 | 0.117 | - | 0.546 | 0.428 | 0.551 |

OR-odds ratio; SMD-standardized mean difference; SE –standard error; na-not available

¶ Study has two control groups; *statistically significant p<0.05
